# Gestational Exposure to 10 Classes of Priority Chemicals and Birth Outcomes in the ECHO Cohort

**DOI:** 10.1001/jamanetworkopen.2026.18883

**Published:** 2026-06-17

**Authors:** Jessie P. Buckley, Diana C. Pacyga, Xiaoshuang Xun, Dana Boyd Barr, Emily S. Barrett, Theresa Bastain, Deborah H. Bennett, Joseph M. Braun, Carrie V. Breton, Courtney Carignan, Lisa A. Croen, Anne L. Dunlop, Shohreh F. Farzan, Assiamira Ferrara, Frank D. Gilliland, Julie B. Herbstman, Margaret R. Karagas, Catherine J. Karr, Jordan R. Kuiper, John D. Meeker, Rachel L. Miller, Rachel Morello-Frosch, Thomas G. O’Connor, Jiwon Oh, Frederica P. Perera, Christina A. Porucznik, Megan E. Romano, Sheela Sathyanarayana, Susan L. Schantz, Rebecca J. Schmidt, Allison R. Sherris, Leonardo Trasande, Heather Volk, Deborah J. Watkins, Qi Zhao, Yeyi Zhu, Zhongmin Li, Edo Pellizzari, Kurunthachalam Kannan, Tracey J. Woodruff

**Affiliations:** 1 Department of Epidemiology, Gillings School of Global Public Health, University of North Carolina at Chapel Hill; 2 Department of Epidemiology, Johns Hopkins Bloomberg School of Public Health, Baltimore, Maryland; 3 Gangarosa Department of Environmental Health, Rollins School of Public Health, Emory University, Atlanta, Georgia; 4 Department of Biostatistics and Epidemiology, Environmental and Occupational Health Sciences Institute, Rutgers School of Public Health, Piscataway, New Jersey; 5 Department of Population and Public Health Sciences, Keck School of Medicine, University of Southern California, Los Angeles; 6 School of Public Health, Washington University in St Louis, St Louis, Missouri; 7 Department of Public Health Sciences, University of California, Davis; 8 Department of Epidemiology, Brown University, Providence, Rhode Island; 9 Department of Food Science and Human Nutrition, Michigan State University, East Lansing; 10 Department of Pharmacology and Toxicology, Michigan State University, East Lansing; 11 Division of Research, Kaiser Permanente Northern California, Pleasanton; 12 Department of Gynecology and Obstetrics, Emory University School of Medicine, Atlanta, Georgia; 13 Department of Environmental Health Sciences, Mailman School of Public Health, Columbia University, New York, New York; 14 Department of Epidemiology, Geisel School of Medicine at Dartmouth, Hanover, New Hampshire; 15 Department of Pediatrics, University of Washington, Seattle; 16 Department of Environmental & Occupational Health Sciences, University of Washington, Seattle; 17 Department of Environmental and Occupational Health, The George Washington University Milken Institute School of Public Health, Washington, DC; 18 Department of Environmental Health Sciences, University of Michigan School of Public Health, Ann Arbor; 19 Division of Clinical Immunology, Icahn School of Medicine at Mount Sinai, New York, New York; 20 School of Public Health, University of California, Berkeley; 21 Department of Environmental Science, Policy and Management, University of California, Berkeley; 22 Department of Psychiatry, University of Rochester, Rochester, New York; 23 Department of Neuroscience, University of Rochester, Rochester, New York; 24 Department of Obstetrics and Gynecology, University of Rochester, Rochester, New York; 25 Department of Family Medicine and Public Health, Spencer Fox Eccles School of Medicine, University of Utah, Salt Lake City; 26 Department of Pediatrics and Seattle Children’s Research Institute, University of Washington, Seattle; 27 Beckman Institute for Advanced Science and Technology, University of Illinois Urbana-Champaign, Urbana; 28 MIND Institute, University of California Davis, Davis; 29 Department of Pediatrics, Grossman School of Medicine, New York University, New York; 30 Department of Population Health, Grossman School of Medicine, New York University, New York; 31 Wagner School of Public Service, New York University, New York; 32 Department of Mental Health, Bloomberg School of Public Health, Johns Hopkins University, Baltimore, Maryland; 33 Department of Preventive Medicine, College of Medicine, University of Tennessee Health Science Center, Memphis; 34 Wadsworth Center, New York State Department of Health, Albany; 35 RTI Fellows Program, Research Triangle Institute, Research Triangle Park, North Carolina; 36 Program on Reproductive Health and the Environment, Department of Obstetrics, Gynecology, and Reproductive Sciences, University of California, San Francisco; 37 Department of Epidemiology and Population Health, School of Medicine, Stanford University, Stanford, California; 38 Woods Institute, Doerr School of Sustainability, Stanford University, Stanford, California

## Abstract

**Importance:**

Gestational environmental chemical exposures are widespread. Some chemicals are known to adversely affect birth outcomes, but many remain understudied.

**Objective:**

To evaluate associations of gestational exposure to a priori identified chemicals in 10 classes with birth outcomes in a large, diverse US cohort.

**Design, Setting, and Participants:**

In the prospective Environmental influences on Child Health Outcomes Cohort study, 5318 mother-child pairs were enrolled from January 1, 2000, to December 31, 2021, with data on gestational urinary chemical concentrations, gestational age at birth, and birth weight. Statistical analysis was performed from January 2024 to February 2026.

**Exposures:**

In single, midgestation (median, 25 weeks [IQR, 21-30 weeks]) urine samples, concentrations of 113 analytes (chemicals or their metabolites) from 10 chemical classes were simultaneously measured: fungicides and herbicides (n = 11), insecticides (n = 20), halogenated phenols (n = 5), organophosphate esters (n = 10), benzophenones (n = 6), bisphenols (n = 14), parabens (n = 6), antimicrobials (n = 2), phthalates or alternative plasticizers (n = 32), and polycyclic aromatic hydrocarbons (PAHs) (n = 7).

**Main Outcomes and Measures:**

Linear mixed-effects regression models with a random effect for site were used to estimate covariate-adjusted differences in gestational age at birth (days) and birth weight-for-gestational age (BW-GA) *z* scores per IQR increase in urinary analyte concentrations. In secondary analyses, odds ratios (ORs) for preterm birth and small for gestational age (SGA) were estimated.

**Results:**

In the sample of 5318 mother-child pairs, most infants (2667 female [50%]; median gestational age at birth, 39.0 weeks [IQR, 38.0-40.0 weeks]) were born to college-educated (67% [3218 of 4785]), parous (56% [2815 of 5007]) mothers (median age at delivery, 30.7 years [IQR, 26.1-34.3 years]). A total of 43 of 113 analytes (38%) were detected in 50% or more of samples. Multiple phthalates or alternative plasticizers were associated with younger gestational age at birth or lower BW-GA *z* scores; for example, summed diisononyl phthalate metabolites were associated with a 0.6-day (95% CI, −1.0 to −0.1 days) younger gestational age (preterm birth OR, 1.16 [95% CI, 1.01-1.34]), and summed phthalate or alternative plasticizers were associated with a 0.06 (95% CI, −0.11 to −0.02) lower BW-GA *z* score (SGA OR, 1.09 [95% CI, 0.93-1.27]). Two halogenated phenols, benzophenone 8, bisphenol F, and several PAHs were associated with lower BW-GA *z* scores; for example, 1- and 9-hydroxphenanthrene were associated with a 0.04 (95% CI, −0.08 to −0.01) lower BW-GA *z* score (SGA OR, 1.13 [95% CI, 1.01-1.27]).

**Conclusions and Relevance:**

This large cohort study of diverse US pregnancies found widespread exposure to 10 classes of environmental chemicals, many of which were associated with differences in gestational age at birth or lower BW-GA *z* scores. These findings indicate that reducing gestational exposure to chemicals, particularly phthalates or alternative plasticizers and PAHs, could promote healthy deliveries and better child outcomes.

## Key Points

**Question:**

Are gestational exposures to 10 classes of widely used chemicals associated with younger gestational age at birth or lower birth weight?

**Findings:**

In this cohort study measuring 113 analytes in 5318 maternal gestational urine samples, multiple phthalates or alternative plasticizers and polycyclic aromatic hydrocarbons were associated with younger gestational age at birth or lower birth weight *z* scores.

**Meaning:**

This study indicates that reducing gestational exposure to chemicals, particularly phthalates or alternative plasticizers and polycyclic aromatic hydrocarbons, could improve birth outcomes and future child health.

## Teaser

This cohort study evaluates associations of gestational exposure to identified chemicals in 10 classes with birth weight and gestational age at birth in a large, diverse US cohort.

## Introduction

Pregnant women in the US are exposed to chemicals via food, water, air, dust, and consumer and personal care products.^1,2^ Chemicals cross the placenta, resulting in direct fetal exposure, which can increase the risk of adverse reproductive and developmental outcomes.^3,4^ Gestational age at birth and birth weight are known factors associated with neonatal morbidity and mortality^5,6^ that can be influenced by gestational chemical exposures.^7,8,9,10,11^ Although more than 1100 pesticidal active ingredients are registered for use^12^ and more than 40 000 synthetic chemicals are allowed for use in the US,^13^ health effects are unstudied for most chemicals,^14^ and prior investigations have typically focused on a few well-studied chemical classes.

We deployed an innovative analytical chemistry method^15^ to measure 113 analytes from 10 chemical classes in pregnancy urine samples and assess their associations with birth outcomes in a large cohort study drawn from the National Institutes of Health Environmental influences on Child Health Outcomes (ECHO) Cohort. We identified selected chemicals a priori via a rigorous and structured process that prioritized those with high opportunity for exposure and high probability of adverse birth and child health outcomes.^14^ We hypothesized that higher gestational exposures to chemicals in multiple classes that had been prioritized based on their potential to harm development would be associated with altered gestational age at birth and birth weight-for-gestational age (BW-GA) *z* scores.

## Methods

The ECHO Cohort is a prospective cohort study comprising more than 60 000 US children.^16,17^ Pregnant women provided written informed consent, and study protocols for cohort sites were approved by the local or central ECHO institutional review boards. The Johns Hopkins University Bloomberg School of Public Health institutional review board approved the involvement of the ECHO Data Analysis Center. We followed the Strengthening the Reporting of Observational Studies in Epidemiology (STROBE) reporting guideline for cohort studies.

### Study Participants

ECHO Cohort sites were given the opportunity to provide midpregnancy (median, 25 weeks [IQR, 21-30 weeks]) urine samples (1 per pregnancy) for quantification of chemical concentrations using a multiclass chemical panel. Due to budget constraints, sites selected participants based on urine availability and their own criteria (eg, participants with child follow-up). In total, 21 sites provided urine samples from 6107 of 20 584 women. We excluded women with no measure of urine dilution (n = 8); women whose child’s sex was unknown (n = 200); women whose child’s birth weight was unknown (n = 353); women whose child’s gestational age was unknown or longer than 42 weeks, necessary to calculate BW-GA *z* scores^18^ (n = 8); women carrying twins (n = 32); women from 3 sites with fewer than 50 participants each (n = 132); and women with urine samples collected at or after birth or with unknown timing (n = 56) (eFigure 1 in Supplement 1). For women with 2 eligible children (27 children), we excluded a randomly selected child (eFigure 1 in Supplement 1). Our final sample included 5318 women with singleton infants from 18 sites enrolled from January 1, 2000, to December 31, 2021 (eTable 1 in Supplement 1).

### Urinary Chemical Biomarker Assays

ECHO Cohort sites shipped frozen urine samples on dry ice to the Wadsworth Center Human Health Exposure Analysis Resource laboratory^19^ for analysis. Using solid-phase extraction coupled with high-performance liquid chromatography–tandem mass spectrometry,^15,20^ urinary concentrations of 113 analytes, representing parent chemicals or their metabolites, from 10 chemical classes were quantified simultaneously: fungicides and herbicides (n = 11), insecticides (n = 20), halogenated phenols (n = 5), organophosphate esters (n = 10), benzophenones (n = 6), bisphenols (n = 14), parabens (n = 6), antimicrobials (n = 2), phthalates and alternative plasticizers (eg, metabolites of cyclohexane-1,2-dicarboxylic acid-diisononyl ester [DiNCH], terephthalates) (n = 32), and polycyclic aromatic hydrocarbons (PAHs) (n = 7). Descriptions of the sample randomization, analytical chemistry methods, quality control analysis^21^ and results, and methods for values below the detection limits are provided in the eAppendix and eTables 16 to 19 in Supplement 2. Analyte names and acronyms are provided in eTable 2 in Supplement 1.

### Measures of Gestational Age at Birth and Birth Weight

The ECHO Data Analysis Center harmonized data for gestational age at birth and birth weight collected by each site, and we calculated sex-specific BW-GA *z* scores for all infants using an external US reference population.^18^ For secondary analyses, we categorized gestational age at birth (completed weeks) as preterm (<37 weeks), early term (37-38 weeks), full term (38-40 weeks), and late term (41-42 weeks). We categorized BW-GA as small for gestational age (SGA; BW-GA <10th percentile), appropriate for gestational age (AGA; 10th-90th percentile), or large for gestational age (LGA; BW-GA >90th percentile).

### Covariate Measures

We used harmonized covariate information to adjust for maternal age (continuous), participant-reported race and ethnicity (categorical; Hispanic, non-Hispanic Asian, non-Hispanic Black, non-Hispanic White, and other [American Indian or Alaska Native, Native Hawaiian or Other Pacific Islander, multiple races or ethnicities, or those who selected “other race”]), prepregnancy body mass index (continuous), education (categorical), parity (binary), season of urine sample collection (categorical), year of urine sample collection (continuous), and self-reported tobacco use during pregnancy (binary) and to explore effect measure modification (EMM) by newborn sex assigned at birth (binary). We adjusted for race and ethnicity as social constructs given observed racial and ethnic disparities in chemical exposures and birth outcomes.

### Statistical Analysis

Statistical analysis was performed from January 2024 to February 2026. For each chemical class, we calculated a cumulative sum measure of exposure to all analytes within the class (eTable 2 in Supplement 1) by dividing each analyte concentration by its molar weight and summing the molar concentrations. We adjusted analyte concentrations for urinary dilution using specific gravity^22,23^ and applied log_2_ transformation to improve model fit.^24^ We calculated Spearman correlation coefficients among analytes detected in 50% or more of participants and plotted them with a heat map. We calculated Spearman correlations between analytes and categorical covariates by first transforming variables into numeric factors, and we calculated biserial correlations between analytes and binary variables. We visualized these correlations using a chord diagram (R package circlize)^25^ and plotted correlations of 0.1 or more.

We modeled analytes with 70% or greater detection frequency and molar sums as continuous log_2_-specific gravity-adjusted variables scaled by their IQRs (eTable 2 in Supplement 1) to facilitate comparison of analyte effect sizes. Given our large sample size, we modeled analytes with 5% to less than 70% detection frequency as binary variables (detected vs not detected). We did not model analytes with less than 5% detection frequency, considering limited statistical power. We estimated differences and 95% CIs for continuous gestational age at birth and BW-GA *z* scores using linear mixed-effects regression models including site as a random effect to account for site-level clustering (R package lme4).^26^ For adjusted models, we imputed missing covariate values with multiple imputation by chained equations using 10 imputed datasets each with 5 iterations (R package mice)^27^ and adjusted for the covariates described.

In secondary analyses, we estimated odds ratios (ORs) and 95% CIs using multinomial mixed-effects regression for categorical gestational age (preterm, early-term, and late-term births compared with full-term births) and logistic mixed-effects regression for categorical BW-GA (SGA or LGA compared with AGA; R package lme4).^26^ We also evaluated EMM by newborn sex in stratified models and calculated EMM *P* values in models including an interaction term between analyte and sex.

We conducted 4 sensitivity analyses. First, we examined potential nonlinear dose-response associations for analytes with 50% or greater detection frequency. We created quartiles if the analyte was detected in 75% or more of participants; otherwise, values below the limit of detection were assigned to the lowest category, and we created 3 equal-sized categories among participants with values at or above the limit of detection. Second, we evaluated each site’s influence on associations by conducting leave-1-site-out analyses excluding participants from 1 site at a time. Third, we assessed potential copollutant confounding by jointly including all chemical class molar sums in the same model. Fourth, although this is a hypothesis-driven study, we applied the Benjamini-Hochberg procedure within each primary outcome (overall and separately by sex) to control the false discovery rate (FDR) for the number of tests (n = 108).

We applied a 2-sided α level of .05 for statistical significance of main effects and EMM. We conducted all analyses using R statistical software, version 4.4.0 (R Project for Statistical Computing).

## Results

### Descriptive Results

In our analytic sample of 5318 mother-child pairs, the median gestational age at birth was 39.0 weeks (IQR, 38.0-40.0 weeks), with 2667 females (50%) and 2651 males (50%) (Table). The median maternal age at delivery was 30.7 years (IQR, 26.1-34.3 years) and most mothers were college educated (67% [3218 of 4785]) and had at least 1 prior child (56% [2815 of 5007]). The sample was 24% Hispanic (1273 of 5260), 5% non-Hispanic Asian (264 of 5260), 19% non-Hispanic Black (999 of 5260), 48% non-Hispanic White (2537 of 5260), and 4% other race or ethnicity (187 of 5260). The maternal characteristics of the included women were similar to those of all women from the participating sites (eTable 3 in Supplement 1). A total of 390 newborns (7%) were delivered preterm, 661 (12%) were late term, 562 (11%) were SGA, and 590 (11%) were LGA.

**Table.** Characteristics of Newborns and Their Mothers

Characteristic	No. (%) (N = 5318)
**Newborn characteristics**	
Sex	
Male	2651 (49.8)
Female	2667 (50.2)
Gestational age at birth	
Preterm	390 (7.3)
Early term	480 (9.0)
Full term	3787 (71.2)
Late or postterm	661 (12.4)
Size for gestational age	
Appropriate for gestational age	4166 (78.3)
Large for gestational age	590 (11.1)
Small for gestational age	562 (10.6)
Gestational age at birth, median (IQR), wk	39.0 (38.0 to 40.0)
Birth weight, median (IQR), g	3355 (3030 to 3690)
Birth weight, median (IQR), *z* score	0.0 (−0.7 to 0.7)
**Maternal characteristics^a^**	
Self-reported race and ethnicity	
Hispanic	1273 (24.2)
Non-Hispanic Asian	264 (5.0)
Non-Hispanic Black	999 (19.0)
Non-Hispanic White	2537 (48.2)
Other^b^	187 (3.6)
Education	
High school degree or less	1567 (32.7)
College degree	2100 (43.9)
Graduate or professional degree	1118 (23.4)
Parity	
≥1 Child	2815 (56.2)
No child	2192 (43.8)
Tobacco use during pregnancy	
No	4022 (93.0)
Yes	304 (7.0)
Season of urine sample collection	
Autumn (September-November)	1241 (23.3)
Winter (December-February)	1243 (23.4)
Spring (March-May)	1435 (27.0)
Summer (June-August)	1399 (26.3)
Year of urine sample collection	
2000-2010	1071 (20.1)
2011-2015	1190 (22.4)
2016-2018	1695 (31.9)
2019-2021	1362 (25.6)
Site (location)	
ARCH (Michigan)	176 (3.3)
ATLANTA (Georgia)	113 (2.1)
CIOB (California)	568 (10.7)
M and N (New York)	271 (5.1)
CANDLE (Tennessee)	692 (13.0)
FAIR START (New York)	240 (4.5)
I-KIDS (Illinois)	296 (5.6)
MINNIE (Colorado)	81 (1.5)
MAGEE (Pennsylvania)	86 (1.6)
MADRES (California)	212 (4.0)
MARCH (Michigan)	569 (10.7)
NHBCS (New Hampshire)	628 (11.8)
PETALS (California)	139 (2.6)
ROCHESTER (New York)	261 (4.9)
SIBLING (New York)	95 (1.8)
GAPPS (Washington)	300 (5.6)
NYU CHES (New York)	448 (8.4)
UCP (Utah)	143 (2.7)
Age at delivery, median (IQR), y	30.7 (26.1 to 34.3)
Prepregnancy BMI, median (IQR)	25.4 (22.2 to 30.5)
Gestational age at urine collection, median (IQR), wk	25.0 (21.0 to 30.0)

Abbreviations: ARCH, Archive for Research in Child Health; ATLANTA, Atlanta ECHO Cohort of Emory University; BMI, body mass index (calculated as weight in kilograms divided by height in meters squared); CANDLE, Conditions Affecting Neurocognitive Development and Learning in Early Childhood; CIOB, Chemicals in Our Bodies; FAIR START, Columbia Center for Children’s Environmental Health—Fair Start Cohort; GAPPS, The Global Alliance to Prevent Prematurity and Stillbirth; I-KIDS, Illinois Kids Development Study; M and N, Columbia Center for Children’s Environmental Health–Mothers and Newborns; MADRES, Maternal and Development Risks from Environmental and Social Stressors; MAGEE, The MAGEE Cohort Study; MARCH, Michigan Archive for Research in Child Health; MINNIE, Maternal and Infant Nutrition, Neurodevelopment, and In utero Exposures; NHBCS, New Hampshire Birth Cohort Study; NYU CHES, The NYU Children’s Health and Environment Study; PETALS, Pregnancy Environment and Lifestyle Study; ROCHESTER, The Rochester Cohort Study; SIBLING, Columbia Center for Children’s Environmental Health Mothers and Newborns—Sibling Study; UCP, Utah Children’s Project.

^a^ Missing: maternal race and ethnicity (n = 58), education (n = 533), parity (n = 311), tobacco use (n = 992), prepregnancy BMI (n = 441).

^b^ Includes mothers who identified as American Indian or Alaska Native, Native Hawaiian or Other Pacific Islander, multiple races or ethnicities, or those who selected “other race” but provided no further details.

**Figure 1.**
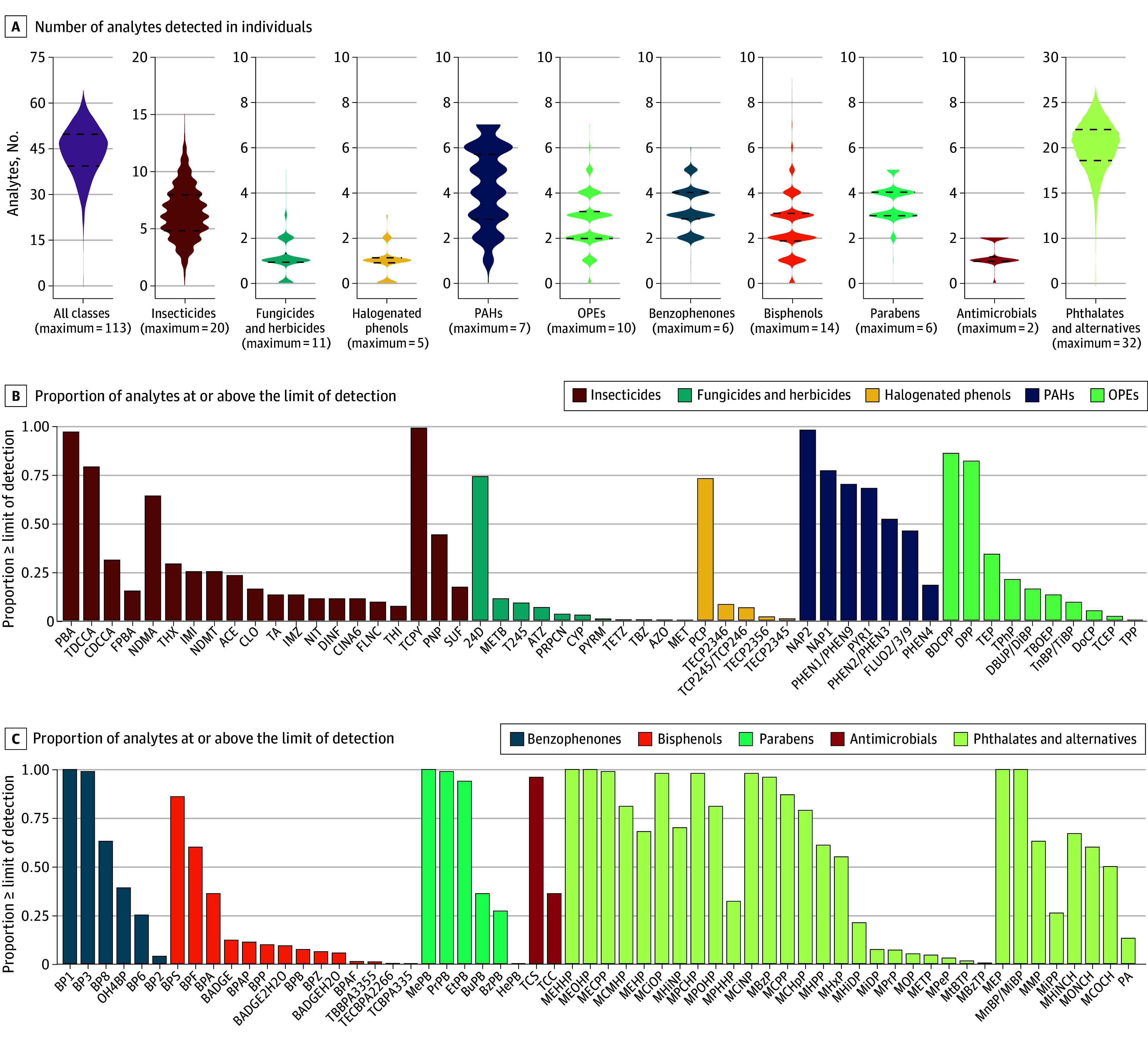
Violin Plots and Bar Graphs for Detection of Analytes in the Study Samples A, Distributions of the number of analytes detected in individuals. B, Proportion of insecticides, fungicides and herbicides, halogenated phenols, polycyclic aromatic hydrocarbons (PAHs), and organophosphate esters (OPEs) at or above the limit of detection. C, Proportion of benzophenones, bisphenols, parabens, antimicrobials, and phthalates and alternatives at or above the limit of detection. Within the violin plots, the black filled circle indicates the median, while the lower and upper dashed lines represent the 25th and 75th percentiles, respectively. Analyte acronyms are defined in eTable 2 in Supplement 1.

**Figure 2.**
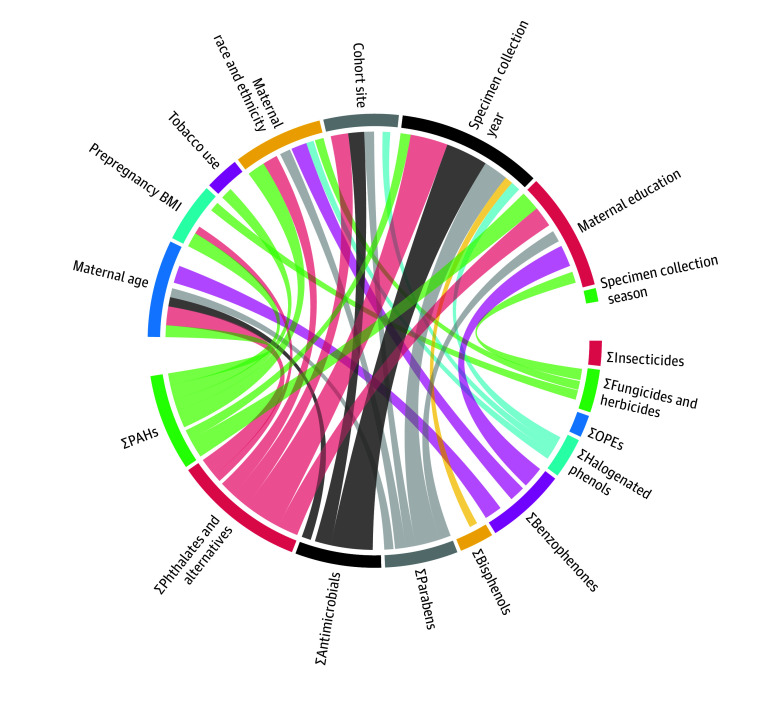
Chord Diagram of Correlations Between Chemical Class Sums and Covariates Lines between covariates and chemical class sums represent correlations of 0.1 or more. Wider lines represent stronger correlations ranging from 0.1 to 0.6. BMI indicates body mass index; OPEs, organophosphate esters; and PAHs, polycyclic aromatic hydrocarbons. Σ indicates cumulative molar sum.

Of the 113 analytes (chemicals or their metabolites), 110 (97%) were detected in at least 1 sample, 60 (53%) were detected in 5% to less than 70% of samples, and 31 (27%) were detected in 70% or more of samples (Figure 1; eTable 2 in Supplement 1). Forty-three analytes (38%) were detected in 50% or more of samples, the median number of analytes detected per participant was 45 (IQR, 40-50), and the maximum number of analytes detected in a sample was 64 (Figure 1A). At least 1 analyte in every class was detected in 70% or more of samples, with universal detection of benzophenone-1 (BP1), methyl paraben (MePB), mono (2-ethyl-5-hydroxyhexyl) phthalate (MEHHP), mono (2-ethyl-5-oxohexyl) phthalate (MEOHP), monoethyl phthalate (MEP), and mono-n-butyl phthalate and mono-isobutyl phthalate (MnBP/MiBP) (Figure 1B and C). Correlations between chemical classes were weak to moderate (eFigure 2 in Supplement 1). Some correlations were stronger within chemical classes, for example, among pyrethroids (3-phenoxybenzoic acid [PBA] and trans-3-(2,2-dichlorovinyl)-2,2-dimethylcyclopropane carboxylic acid [TDCCA]), benzophenones (BP1 and benzophenone-3 [BP3]), parabens (MePB and propyl paraben [PrPB]), and metabolites of a given parent phthalate (*r* > 0.7). Metabolites of summed DiNCH (alternative plasticizer) were weakly inversely correlated with most phthalate metabolites. Specimen collection year was correlated with most chemical class sums (Figure 2), with decreasing trends over time for summed halogenated phenols, summed bisphenols, summed parabens, summed antimicrobials, and summed phthalates and alternatives and increasing trends for summed PAHs (eTables 4 and 5 in Supplement 1). Despite decreasing trends or no trend for their class-based sums, certain neonicotinoid insecticides (*N*-desmethyl acetamiprid [NDMA], thiamethoxam [THX]), bisphenols (bisphenol S [BPS]), and phthalates or alternative plasticizers (mono-2-[propyl-6-oxoheptyl]-phthalate [MPOHP], mono-2-[propyl-6-hydroxy-heptyl]-phthalate [MPHHP], monohydroxy-iso-decyl phthalate [MHiDP], and summed DiNCH) showed increasing trends (eTables 4 and 5 in Supplement 1).

### Associations With Gestational Age at Birth

**Figure 3.**
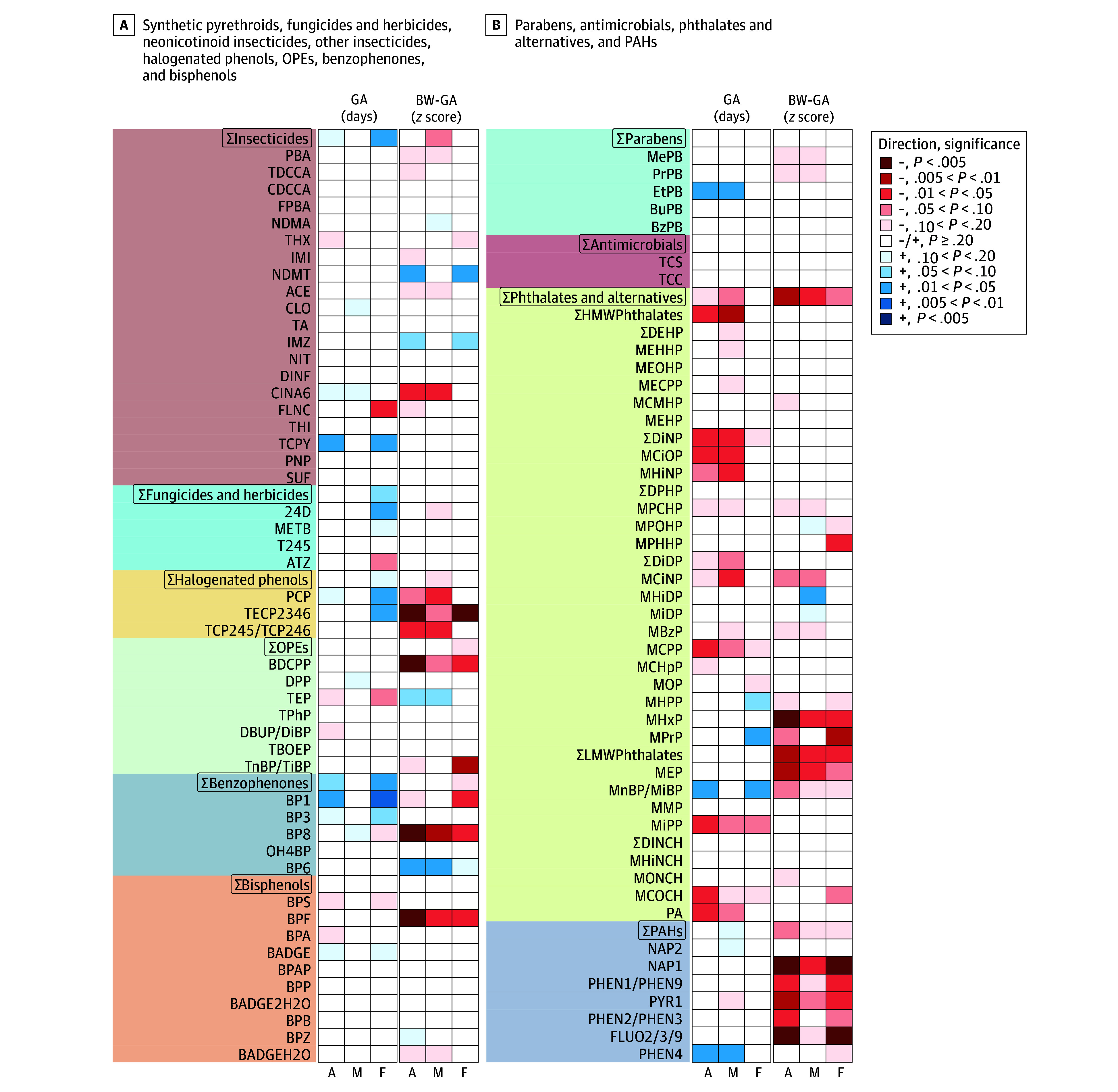
Heat Maps Showing Associations With Gestational Age (GA) at Birth and Birth Weight for Gestational Age (BW-GA) *z* Scores A, Associations of synthetic pyrethroids, fungicides and herbicides, neonicotinoid insecticides, other insecticides, halogenated phenols, organophosphate esters (OPEs), benzophenones, and bisphenols with GA at birth and BW-GA *z* scores. B, Associations of parabens, antimicrobials, phthalates and alternatives, and polycyclic aromatic hydrocarbons (PAHs) with GA at birth and BW-GA *z* scores. Σ indicates cumulative molar sum. The shading represents the strength of the *P* value: the smaller the *P* value, the darker (red or blue) the rectangle. The plus sign denotes a positive direction of association, and the minus sign denotes a negative direction of association. Linear mixed-effects regression models accounted for site as a random effect, maternal age, prepregnancy body mass index, education, parity, season of urine sample collection, year of urine sample collection, tobacco use, and race and ethnicity. Numeric results can be found in eTable 6 in Supplement 1 for GA at birth and eTable 8 in Supplement 1 for BW-GA *z* scores. Analyte acronyms are defined in eTable 2 in Supplement 1. A indicates all newborns; F, female newborns; and M, male newborns.

**Figure 4.**
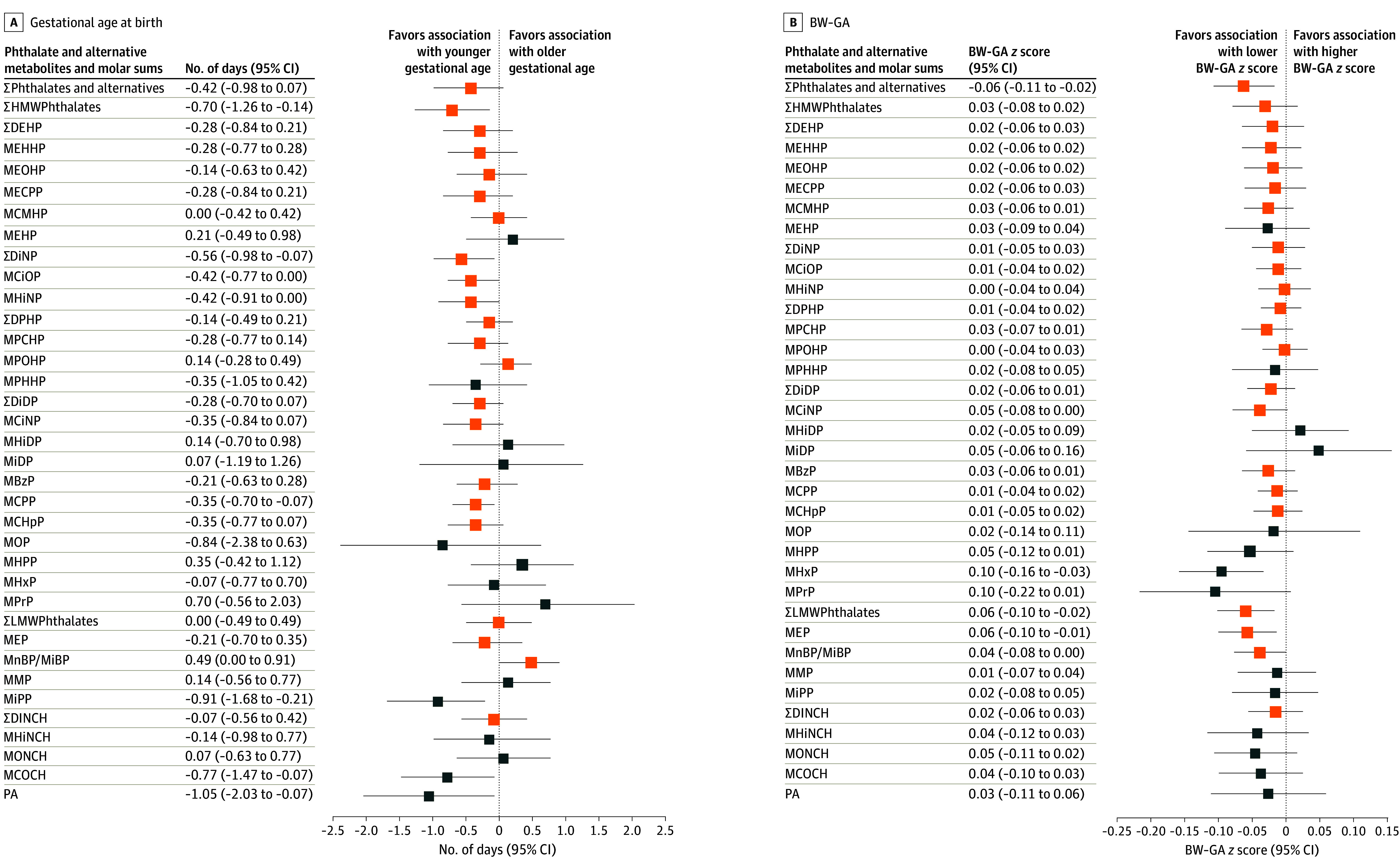
Forest Plots Showing Associations of Phthalate Metabolites and Molar Sums With Continuous Gestational Age at Birth and Birth Weight for Gestational Age (BW-GA) *z* Scores A, Gestational age at birth. B, BW-GA *z* scores. Data are presented as the day or *z* score difference (squares) and 95% CIs (horizontal lines) in gestational age at birth (days) or BW-GA (*z* score), respectively. The orange squares represent analytes modeled per IQR increase in concentration; the blue squares represent analytes modeled as binary variables (detected vs not detected). Σ indicates cumulative molar sum. Linear mixed-effects regression models accounted for site as a random effect and adjusted for maternal age, race and ethnicity, prepregnancy body mass index, education, parity, season of urine collection, year of urine collection, and tobacco use. Estimates are reported in eTables 6 and 8 in Supplement 1. Analyte acronyms are defined in eTable 2 in Supplement 1.

In adjusted models, 8 analytes or sums were significantly associated with younger gestational age at birth, while 6 analytes or sums were associated with older gestational age at birth (Figure 3; eTable 6 in Supplement 1). All 8 analytes or sums associated with younger gestational age were metabolites of phthalates and alternative plasticizers (Figure 4). For example, each IQR increase in summed diisononyl phthalate (DiNP) metabolites and detection of phthalic acid (PA) were associated with a 0.6-day (95% CI, −1.0- to −0.1-day) and 1.1-day (95% CI, −2.0- to −0.1-day) younger gestational age at birth, respectively (Figure 4). Conversely, summed neonicotinoids (β per IQR increase, 0.42 [95% CI, 0.00-0.84] with strongest contributors dinotefuran [DINF] and 6-chloronicotinic acid [CINA6]), the insecticide 3,5,6-trichloro-2-pyridinol (TCPY; β per IQR increase, 0.42 [95% CI, 0.00-0.91]), the benzophenone BP1 (β per IQR increase, 0.56 [95% CI, 0.07-1.05]), the paraben ethyl paraben (EtPB; β per IQR increase, 0.49 [95% CI, 0.07-0.84]), the phthalate metabolite MnBP/MiBP (β per IQR increase, 0.49 [95% CI, 0.00-0.91]), and the PAH 4-hydroxyphenanthrene (PHEN4; β for detected vs not detected, 0.91 [95% CI, 0.07-1.75]) were associated with older gestational age at birth (Figure 3).

In secondary analyses of categorical outcomes (eTable 7 in Supplement 1), results were consistent with primary analyses, with summed DiNP (OR per IQR increase, 1.16 [95% CI, 1.01-1.34]), mono (3-carboxypropyl) phthalate (MCPP; OR per IQR increase, 1.16 [95% CI, 1.03-1.30]), and PA (OR for detected vs not detected, 1.48 [95% CI, 1.11-1.98]) associated with higher odds of preterm birth. In addition, the neonicotinoid insecticide NDMA was associated with preterm birth (OR for detected vs not detected, 1.28 [95% CI, 1.01-1.62]). Similar to the primary findings, the benzophenone BP1 (OR per IQR increase, 0.82 [95% CI, 0.69-0.97]), the paraben EtPB (OR per IQR increase, 0.84 [95% CI, 0.73-0.97), the phthalate MnBP/MiBP (OR per IQR increase, 0.84 [95% CI, 0.73-0.96]), and the PAHs 1-hydroxphenanthrene and 9-hydroxyphenanthrene ([PHEN1/PHEN9]; OR per IQR increase, 0.88 [95% CI, 0.77-0.99]) were associated with lower odds of preterm birth.

In secondary analyses of sex differences, associations of phthalate and alternative plasticizer metabolites with younger gestational age at birth and higher odds of preterm birth were generally stronger among male than female newborns (Figure 3; eTables 6 and 7 in Supplement 1). The neonicotinoid insecticide NDMA and the alternative plasticizer cyclohexane-1,2-dicarboxylic acid mono hydroxyisononyl ester (MHiNCH) were associated with higher odds of preterm birth in females but not males (eTable 7 in Supplement 1). Some analytes and sums were associated with older gestational age at birth or lower odds of preterm birth among females but not males (summed fungicides and herbicides, 2,4-dichlorophenoxyacetic acid [24D], summed other insecticides, TCPY, pentachlorophenol [PCP], 2,3,4,6-tetrachlorophenol [TECP2346], summed benzophenones, BP1, BP3, triclosan [TCS], MnBP/MiBP, mono-2-heptyl phthalate [MHPP], and mono-propyl phthalate [MPrP]; Figure 3; eTables 6 and 7 in Supplement 1).

### Associations With BW-GA *z* Scores

In adjusted models, 15 analytes or sums were significantly associated with lower BW-GA *z* scores, while 2 were associated with higher BW-GA *z* scores (Figure 3; eTable 8 in Supplement 1). Four phthalate and alternative plasticizer analytes or sums and 6 PAH analytes or sums were associated with lower BW-GA *z* scores; IQR increases in summed phthalates and alternatives and summed PAHs were associated with −0.06 (95% CI, −0.11 to −0.02) and −0.04 (95% CI, −0.08 to 0.00) lower BW-GA *z* scores, respectively (Figure 3; eTable 8 in Supplement 1). Within these classes, associations with lower BW-GA *z* scores were observed for MEP (β per IQR increase, –0.06 [95% CI, –0.10 to –0.02]); MHxP (β for detected vs not detected, –0.10 [95% CI, –0.16 to –0.03]); NAP1 (β per IQR increase, –0.06 [95% CI, –0.10 to –0.03]); PHEN1/PHEN9 (β per IQR increase, –0.04 [95% CI, –0.08 to –0.01]); PYR1 (β for detected vs not detected, –0.09 [95% CI, –0.15 to –0.03]); PHEN2/PHEN3 (β for detected vs not detected, –0.06 [95% CI, –0.12 to –0.00]); and 2-hydroxyfluorene, 3-hydroxyfluorene, and 9-hydroxyfluorene (FLUO2/3/9; β for detected vs not detected, –0.12 [95% CI, –0.18 to –0.06]). Other analytes associated with lower BW-GA *z* scores included the neonicotinoid insecticide CINA6 (β for detected vs not detected, −0.09 [95% CI, −0.18 to −0.01]); the halogenated phenols TECP2346 (β for detected vs not detected, −0.18 [95% CI, −0.28 to −0.08]) and 2,4,5-trichlorophenol and 2,4,6-trichlorophenol (TCP245/TCP246; β for detected vs not detected, −0.13 [95% CI, −0.24 to −0.01]); the organophosphate ester bis(1,3-dichloro-2-propyl) phosphate (BDCPP; β per IQR increase, −0.05 [95% CI, −0.09 to −0.02]); the benzophenone benzophenone-8 (BP8; β for detected vs not detected, −0.10 [95% CI, −0.16 to −0.04]); and the bisphenol bisphenol F (BPF; β for detected vs not detected, −0.09 [95% CI, −0.14 to −0.03]) (Figure 3). Conversely, the neonicotinoid insecticide *N*-desmethyl thiamethoxam (NDMT; β for detected vs not detected, 0.07 [95% CI, 0.00-0.13]) and the benzophenone benzophenone-6 (BP6; β for detected vs not detected, 0.08 [95% CI, 0.02-0.15]) were associated with higher BW-GA *z* scores (Figure 3).

In secondary analyses of categorical outcomes, the findings were consistent with the continuous BW-GA *z* score results, although the 95% CIs were wider (eTable 9 in Supplement 1). For example, each IQR increase in summed phthalates and alternatives was associated with 1.09 (95% CI, 0.93-1.27) times higher odds of SGA. The neonicotinoid insecticide nitenpyram (NIT; OR for detected vs not detected, 1.34 [95% CI, 1.03-1.76]) and the PAH PHEN1/PHEN9 (OR per IQR increase, 1.13 [95% CI, 1.01-1.27]) were associated with higher odds of SGA compared with AGA, while the benzophenones 4-hydroxybenzophenone (OH4BP; OR for detected vs not detected, 0.80 [95% CI, 0.66-0.98]) and BP6 (OR for detected vs not detected, 0.70 [95% CI, 0.55-0.88]) were associated with lower odds of SGA (eTable 9 in Supplement 1). Several analytes in most chemical classes were associated with lower odds of LGA (eTable 9 in Supplement 1).

In secondary analyses, sex differences were observed for several analytes: summed neonicotinoids (insecticide) were associated with lower BW-GA *z* scores among males only, MPHHP (phthalate) was associated with lower BW-GA *z* scores among females only, and MHiDP (phthalate) was associated with higher BW-GA *z* scores among males only (Figure 3; eTable 8 in Supplement 1). Associations with SGA were generally stronger among males than females (eTable 9 in Supplement 1).

### Sensitivity Analyses

In analyses using analyte categories, compared with the first category (reference group), associations were generally strongest in the highest category or across the second, third, and fourth categories for both gestational age at birth (eTable 10 in Supplement 1) and BW-GA *z* scores (eTable 11 in Supplement 1). In contrast, some analytes showed nonlinear associations, including 6 analytes (MePB, MEOHP, mono ethyl hexyl phthalate [MEHP], mono-carboxy isononyl phthalate [MCiNP], mono-hexyl phthalate [MHxP], and cyclohexane-1,2-dicarboxylic acid mono carboxyisooctyl ester [MCOCH]) with gestational age and 11 analytes (PBA, TDCCA, BP3, BPF, PrPB, MEOHP, MCiNP, MEP, MnBP/MiBP, mono-methyl phthalate [MMP], and cyclohexane-1,2-dicarboxylic acid-mono[oxo-isononyl] ester [MONCH]) with BW-GA *z* scores. In leave-1-site-out analyses, effect sizes did not appreciably differ when omitting 1 site at a time for gestational age at birth (eTable 12 in Supplement 1) or BW-GA *z* scores (eTable 13 in Supplement 1). We observed similar effect estimates, with slightly wider 95% CIs, when including all chemical class sums (*r* < 0.55 between sums) (eFigure 2 in Supplement 1) in a single model to account for potential coexposure confounding (eTables 14 and 15 in Supplement 1). Finally, associations of TECP2346, BP8, 1-hydroxynaphthalene (NAP1), and FLUO2/3/9 with BW-GA *z* scores remained after FDR correction (eTables 6 and 8 in Supplement 1).

## Discussion

We found ubiquitous exposure to 10 classes of environmental chemicals in this prospective cohort study of 5318 US pregnancies. Of the 113 chemicals or metabolites measured, the median number of analytes detected per woman was 45. Multiple phthalate and alternative plasticizer metabolites, including understudied and newer alternatives for phthalates, were associated with younger gestational age at birth, lower BW-GA *z* scores, and higher risk of preterm birth, with 95% CIs that did not cross the null. PAHs, some halogenated phenols (TECP2346 and TCP245/TCP246), BP8, and BPF were associated with lower BW-GA *z* scores. Conversely, certain insecticides and benzophenones were associated with older gestational age at birth, higher BW-GA *z* scores, or lower risk of preterm birth or SGA. Given that chemical exposures are widespread, small individual-level reductions in gestational age or birth weight can result in meaningful population-level increases in the number of infants born preterm or with low birth weight, particularly in vulnerable populations with a higher baseline risk of these adverse birth outcomes.^28^

We observed widespread chemical exposures during pregnancy, with ubiquitous detection (≥70% of participants) of 31 analytes from 10 chemical classes. As urinary analytes are relatively short lived in the body, our results demonstrate that pregnant women are continuously exposed to many chemicals from a range of sources, including ambient and indoor air, house dust, personal care products, building materials, and food.^14^ Multiple analytes were detected in samples from nearly every woman, and we observed increasing temporal trends for several analytes used as alternatives to other chemicals of concern, including BPS^29^ and the alternative plasticizer DiNCH.^30,31^ We observed near-universal detection of TCPY, a metabolite of the neurotoxic organophosphorus pesticide chlorpyriphos,^32^ and frequent detection of several neonicotinoid insecticide analytes, with increasing trends over time. Metabolites of PAHs, emitted from traffic and wood smoke and found in certain foods, such as meats,^33^ were also widely detected.

We found the strongest associations with gestational age and birth weight for biomarkers of understudied compounds in several chemical classes (phthalates: DiNP, DiDP, and DNHxP metabolites; benzophenones: BP1, BP6, and BP8; bisphenols: BPF; halogenated phenols: TECP2346 and TCP245/TCP246). This finding highlights both concerns about toxic substitutions^34^ and the importance of expanding research beyond chemicals that have been highly studied, such as DEHP (phthalate),^35,36,37,38,39,40,41^ BP3 (benzophenone),^11^ and BPA (bisphenol).^11,35,42,43,44,45^ Our findings illustrate that exposure to understudied but commonly used chemicals during pregnancy can have adverse health effects, emphasizing the importance of generating health data for chemicals to which the public is exposed. For example, the DnHxP metabolite MHxP was associated with lower birth weight and higher odds of SGA in our sample. DnHxP is structurally similar to other phthalates known to influence pregnancy outcomes, and the state of California and the National Toxicology Program have identified it as a reproductive and/or developmental toxicant.^46,47^ Higher concentrations of MHxP have been associated with lower odds of a live birth among couples undergoing in vitro fertilization^48^ as well as more externalizing problems^49^ and lower IQ^50^ in childhood. In addition, the results for summed DiDP and summed DiNP are relevant to a recent US Environmental Protection Agency health review of these phthalates for determining appropriate interventions to prevent harmful exposures; the review did not fully evaluate human evidence for preterm birth and did not identify this end point in their analysis for “unreasonable risk of injury to human health,” including developmental toxic effects.^51,52^

Our results for phthalates and PAHs were consistent with high-quality systematic reviews and meta-analyses that determined phthalates increased risk of preterm birth and lower gestational age at birth^9,38^ and lower birth weight^40^ and that PAHs decrease birth weight.^53,54^ We observed fewer associations for fungicides and herbicides or insecticides, although some insecticides were associated with older gestational age at birth (TCPY), lower BW-GA *z* score (CINA6), or higher BW-GA *z* scores (NDMT and IMZ). Although numerous studies have examined associations between pesticides and birth outcomes,^8,55,56,57,58^ each focused on a different pesticide subclass, and there is limited research on neonicotinoid insecticides.^8,59^ Among organophosphate esters, BDCPP was associated with lower BW-GA *z* scores, and TEP was associated with higher BW-GA *z* scores; the limited literature on organophosphate esters is similarly mixed.^60^

### Strengths and Limitations

Our study has many strengths. To our knowledge, ours is the largest and most comprehensive study to evaluate the association of exposure to multiple chemical classes with birth outcomes in the US. We studied analytes previously associated with birth outcomes, as well as their potentially toxic alternatives, and we assessed both individual analytes and cumulative exposures to 10 chemical classes. Analytes were simultaneously quantified using a validated, high-throughput method in randomized batches at the same laboratory. We used individual-level data and adjusted for a comprehensive suite of potential confounders. Our cohort included participants from various sites across the US and was diverse in race and ethnicity and socioeconomic position, making our results more generalizable than previous single-cohort studies.

Our analysis also has limitations. Due to their short biological half-lives, a single midpregnancy analyte measurement may not represent usual pregnancy exposure; this misclassification could attenuate effect estimates toward the null.^61^ Long-term urine storage for some ECHO Cohort sites may have influenced measurement of some analytes, but we did not observe differences in leave-1-site-out sensitivity analyses. We measured parent compounds rather than urinary metabolites for some organophosphate esters and pesticides; several parent compounds were frequently detected in urine samples and were associated with birth outcomes, supporting their use as a biomarker of exposure. Similar effect sizes were observed in male and female infants, although subtle sex differences cannot be ruled out. We conducted a hypothesis-driven study, several of our findings are confirmed in other studies, and we applied FDR correction for multiple comparisons. Still, both false-positive and false-negative results are possible.^62^ Finally, we used 10 hypothesis-driven class-based sums to examine cumulative exposures while limiting the number of statistical tests relative to other cumulative mixtures approaches. However, sums assume equal toxic effects of all analytes; future studies could characterize effects of chemicals across classes using other methods.

## Conclusions

In this large, diverse US cohort study, we found widespread exposure of pregnant women to analytes in 10 classes of chemicals, including potentially toxic alternatives for chemicals of concern. Many of the urinary analytes that we measured, particularly phthalates or alternative plasticizers and PAHs, were associated with shorter length of gestation or lower BW-GA *z* scores, indicating adverse effects on growth and development.

## Notes

Supplement 1. **eFigure 1.** Flow Chart of Inclusion/Exclusion Criteria Used to Generate the Analytic Sample **eFigure 2.** Correlations Between Frequently Detected Analyte Concentrations and Chemical Sums **eTable 1.** Environmental influences on Child Health Outcomes (ECHO) Sites Included in the Current Study (18 Sites) **eTable 2.** Distribution of Molar Sum and Individual Analyte Concentrations (n = 5318) **eTable 3.** Characteristics of Newborns and Their Mothers: Comparison of the Study Sample to all ECHO Cohort Participants From 18 Sites **eTable 4.** Distributions of Analyte Concentrations by Sample Characteristics Among Analytes Detected in ≥ 70% of Samples (n = 5318) **eTable 5.** Proportion of Analyte Concentrations Greater Than the Lower Limit of Detection by Sample Characteristics Among Analytes Detected in 5–70 % of Samples (n = 5318) **eTable 6.** Overall and Stratified Associations Between Chemical Biomarker Concentrations and Continuous Gestational Age at Birth (n = 5318) **eTable 7.** Overall and Stratified Associations Between Chemical Biomarker Concentrations and Categorical Gestational Age at Birth (n = 5318) **eTable 8.** Overall and Stratified Associations Between Chemical Biomarker Concentrations and Continuous Birth Weight for Gestational Age z-Scores (n = 5318) **eTable 9.** Overall and Stratified Associations Between Chemical Biomarker Concentrations and Categorical Birth Weight for Gestational Age z-Scores (n = 5318) **eTable 10.** Dose-Response Associations of Analytes Detected in ≥ 50% of Samples With Continuous and Categorical Gestational Age at Birth (n = 5318) **eTable 11.** Dose-Response Associations of Analytes Detected in ≥ 50% of Samples With Continuous and Categorical Birth Weight (n = 5318) **eTable 12.** Associations Between Analytes and Gestational Age Excluding One Cohort Site at a Time **eTable 13.** Associations Between Analytes and Birth Weight for Gestational Age z-scores Excluding One Cohort Site at a Time **eTable 14.** Mutually Adjusted Associations Between Chemical Class Sums and Continuous Gestational Age at Birth (n = 5318) **eTable 15.** Mutually Adjusted Associations Between Chemical Class Sums and Continuous Birth Weight for Gestational Age z-Scores (n = 5318)

Supplement 2. **eAppendix.** Gestational Exposure to Ten Classes of Priority Chemicals and Birth Outcomes in the ECHO Cohort **eTable 16.** Recovery Table for NIST Quality Control Samples **eTable 17.** Summary of Results for Quality Control Spikes **eTable 18.** Summary of Results of HHEAR Quality Control (QC) Pools **eTable 19.** Relative Percent Differences (RPDs) for Analytes Measured in Duplicate Samples from Nine ECHO Cohort Sites
eReferences.

Supplement 3. **Nonauthor Collaborators.** Environmental influences on Child Health Outcomes (ECHO) Cohort Consortium

Supplement 4.
Data Sharing Statement
