# Supplementary material for: Gestational Exposure to 10 Classes of Priority Chemicals and Birth Outcomes in the ECHO Cohort
Source: JAMA Netw Open. 2026 Jun 17;9(6):e2618883. doi: 10.1001/jamanetworkopen.2026.18883 (PMC13276631; doi:10.1001/jamanetworkopen.2026.18883)
Supplement: Supplement 2. — eAppendix. Gestational Exposure to Ten Classes of Priority Chemicals and Birth Outcomes in the ECHO Cohort eTable 16. Recovery Table for NIST Quality Control Samples eTable 17. Summary of Results for Quality Control Spikes eTable 18. Summary of Results of HHEAR Quality Control (QC) Pools eTable 19. Relative Percent Differences (RPDs) for Analytes Measured in Duplicate Samples from Nine ECHO Cohort Sites eReferences. [file jamanetwopen-e2618883-s002.pdf]

Supplementary Online Content

Buckley JP, Pacyga DC, Xun X, et al; Environmental influences on Child Health Outcomes (ECHO) Cohort Consortium. Gestational exposure to 10 classes of priority chemicals and birth outcomes in the ECHO Cohort. *JAMA Netw Open.* 2026;9(6):e2618883. doi:10.1001/jamanetworkopen.2026.18883

eAppendix. Gestational Exposure to Ten Classes of Priority Chemicals and Birth Outcomes in the ECHO Cohort

eTable 16. Recovery Table for NIST Quality Control Samples

eTable 17. Summary of Results for Quality Control Spikes

eTable 18. Summary of Results of HHEAR Quality Control (QC) Pools

eTable 19. Relative Percent Differences (RPDs) for Analytes Measured in Duplicate Samples from Nine ECHO Cohort Sites

eReferences.

This supplementary material has been provided by the authors to give readers additional information about their work.

© 2026 Buckley JP et al. *JAMA Network Open.*

eAppendix. Gestational Exposure to Ten Classes of Priority Chemicals and Birth Outcomes in the ECHO Cohort

Sample randomization

Urine samples were randomized prior to laboratory analysis, with a major goal to minimize cohort site and batch effects. Depending on how many samples were submitted by each cohort site, 1–2% of samples were pulled from each batch of samples so that each batch contained 78 samples from 18 cohort sites. For cohort sites providing a large number of samples (~700–800 samples), about 9 samples were pulled per batch. For cohort sites providing only a small number of samples (<100 samples), only 1 or 2 samples were pulled per batch. Thus, each batch contained samples from all cohort sites, proportionally based on sample size. A total of 82 batches were analyzed for this multiclass chemical project. Each batch of samples also included laboratory QA/QC samples including procedural blanks, blinded duplicate samples, matrix spikes, matrix spike duplicates, standard reference material, and HHEAR QC pools (2 sets one with high concentration and another with low concentration). This randomization scheme was designed to eliminate bias originating from cohort site, batch, and time of analysis.

Analytical chemistry methods

The multiclass method was developed to include as many analytes as possible for chemicals with diverse physiochemical properties. Because metabolites of some chemicals are unknown and standards are not available for some known metabolites, the method included both metabolites and parent compounds with the goal of characterizing exposure to a wide range of chemicals including those prioritized by the ECHO Program.[1] The final selection of biomarkers was based on the performance of the analytes in the method. The performance parameters monitored include recovery, precision, and detection limits. The method achieved optimal performance for 121 chemicals.[2] Of these, 113 met target performance in the current project and were reported.

Briefly, 500 µL of urine samples were fortified with 2.5 ng each of isotope labeled internal standard mixture and then buffered with 500 µL of 1.0 M ammonium acetate (pH 5.5) containing 20 µL of β-

glucuronidase/arylsulfatase (2000 units, Type ALS; Sigma-Aldrich, St. Louis, MO, USA). After gentle mixing, samples were incubated at 37 °C in an incubator shaker for 2 h. After incubation, samples were quenched with 1 mL of 0.145 M sodium phosphate monobasic monohydrate (SPMM, pH 2.0). Samples were then passed through ABS ELUT-Nexus cartridges (60 mg/3 cc; Varian, Walnut Creek, CA, USA) that were conditioned with acetonitrile (1.5 mL) and 0.145 M SPMM (1.5 mL, pH 2.0). After sample loading, cartridges were washed with 0.1 M formic acid (1.5 mL) and 5% MeOH in water (v/v, 1.5 mL) and vacuum dried for 5 min. The target compounds were eluted with acetonitrile (1.0 mL), ethyl acetate (1.0 mL), and 1:1 MeOH:DCM (v/v, 1.0 mL). The eluate was collected into a 15 mL PP tube and concentrated to near-dryness under a gentle nitrogen stream (Organomation Associates Inc., West Berlin, MA, USA). The residue was reconstituted in 250 µL of water:ACN (8:2, v/v), vortexed, briefly centrifuged, and transferred into amber glass vials for liquid chromatography-tandem mass spectrometry (LC-MS/MS) analysis.

Target compounds were analyzed using an ExionLC system (SCIEX, Redwood City, CA, USA) coupled to an AB SCIEX QTRAP 5500 + triple quadrupole mass spectrometer (Applied Biosystems, Foster City, CA, USA) equipped with an electrospray ionization source operated in both positive and negative modes. Chromatographic separation and quantitation of target compounds was achieved by two instrumental methods. The first method involved chromatographic separation of target analytes using an Ultra AQ C18 column (100 mm × 2.1 mm, 3 µm, Restek; Bellefonte, PA, USA) serially connected to a Javelin guard column (Betasil C18, 2.1 mm × 20 mm, 5 µm, Thermo Electron Corp.; Waltham, MA, USA). The HPLC mobile phase comprised 0.1% acetic acid in water (A) and 0.1% acetic acid in MeOH (B). The initial mobile phase composition was 5% B, held for 1 min, then increased to 45% B within 0.2 min, and held for 1.3 min. Then, the composition was increased to 70% B within 2.2 min, then to 99% B in 2.0 min, and held for 3.0 min. Return to initial mobile phase conditions and column equilibration was accomplished in the last 2.3 min, with a total run time of 12 min. The MS/MS method was run in negative ionization mode. Curtain gas (CUR), collision activated dissociation gas (CAD), source temperature (TEM), nebulizer gas (GS1), heater gas (GS2), and turbo ion spray voltage (IS) were set at 25 psi, 10 psi, 550 °C, 50 psi, 70 psi, and -4500 V, respectively.

For the second method, the chromatographic separation of target analytes was achieved using a Betasil™ C18 column (100 mm × 2.1 mm, 5 µm, ThermoFisher; Waltham, MA, USA) serially connected to a Javelin guard column. The HPLC mobile phase comprised water (A) and ACN (B) with the following gradient program; 20% B for 1 min, increased to 60% B within 0.2 min, then to 99% A within 4.3 min, held for 1.5 min, decreased to 20% B

© 2026 Buckley JP et al. *JAMA Network Open.*

within 0.5 min, and equilibrated for 1.5 min, with a total run time of 9 min. The MS/MS analysis was run in both negative and positive ionization modes. CUR, CAD, TEM, GS1, GS2, and IS were set at 20 psi, 8 psi, 600 °C, 60 psi, 70 psi, and -4500/ + 4500 V, respectively.

Target analytes were quantified by isotopic dilution method and a 15-point calibration (at concentrations ranging from 0.01 to 150 ng/mL); a regression coefficient of ≥ 0.99 was used. A pure solvent (MeOH) and a mid-point calibration standard were injected after every 15 samples to check for carryover of target chemicals and instrumental drift in sensitivity.

Several procedural blanks were analyzed to monitor for contamination that can arise from reagents and materials used in sample preparation steps. For each batch of 78 samples, two reagent blanks, matrix blanks, and matrix spikes (purchased urine spiked with native standards at 10 ng for all analytes and IS) were processed. In addition, three replicates of HHEAR quality control (QC) pools, two replicates of CHEAR QC pools, and one of each Standard Reference Material (SRM3672 and SRM3673, NIST, Gaithersburg, MD, USA; IS spiked) containing certified values for several analytes were processed. Trace levels of several analytes were found in reagent blanks, and the concentrations of these blanks were subtracted from the concentrations in the samples.

Due to insufficient resolution to quantify some chemicals individually, we quantified seven analytes that are composites of multiple chemicals: TCP245/TCP246 (a composite of 2,4,5-Trichlorophenol and 2,4,6-Trichlorophenol), DBUP/DiBP (a composite of Dibutyl phosphate and Di-isobutyl phosphate), TnBP/TiBP (a composite of Tri-n-butyl phosphate and Tri-isobutyl phosphate), MnBP/MiBP (a composite of mono-isobutyl phthalate and mono-n-butyl phthalate), PHEN1/PHEN9 (a composite of 1-hydroxphenanthrene and 9-hydroxyphenanthrene), and PHEN2/PHEN3 (a composite of 2-hydroxyphenanthrene and 3-hydroxyphenanthrene), and FLUO2/3/9 (a composite of 2-hydroxyfluorene, 3-hydroxyfluorene, and 9-hydroxyfluorene).

Quality control analysis and results

Data quality was assessed for background contamination, apparent accuracy, and reproducibility of analysis following detail protocols described previously.[3] Reagent blanks were analyzed for background contamination, and these data were used to correct sample concentrations. Apparent accuracy was determined by analyzing National Institute of Standards and Technology (NIST) standard reference materials (SRMs) and QC spikes. Reproducibility was assessed by analyzing QC pool samples and cohort site duplicate sample aliquots.

© 2026 Buckley JP et al. *JAMA Network Open.*

Apparent accuracy: Eighty-two samples each of NIST SRM 3672 and SRM 3673 were run at a rate of one sample per batch. NIST provides reference values of NIST SRM 3672 and SRM 3673 for a subset of the phthalates, parabens, bisphenols, benzophenones, antimicrobials, and polycyclic aromatic hydrocarbons run in this analysis, referred to as target values in the table. Recovery percentages were calculated for analytes with target values. Sixty-two of the analytes measured in NIST SRMs had coefficients of variation (CVs) ≤20%; results near the limit of detection (LOD) are subject to greater uncertainty (eTable 16). In total, 164 matrix spike samples were run at a rate of two samples per batch for this analysis. Overall means and coefficients of variation were calculated for each QC type. Eighty-eight of the analytes measured in QC spikes had CVs ≤ 20%; results near the LOD are subject to greater uncertainty (eTable 17).

Reproducibility: Repeat analysis of two QC pool samples provided an assessment of method extraction and instrument performance. A total of 246 samples each of HHEAR QC pool H-A and H-B were run as statistical field samples at a rate of three samples per batch in the 82 batches in this analysis. Overall means and CVs (%CVs) were calculated for each pool. Sixty-five of the analytes measured in QC pools had CVs ≤20%; results near the LOD are subject to greater uncertainty (eTable 18). Duplicate aliquots of samples were analyzed to determine the reproducibility of sample processing through instrumental analysis (eTable 19). Across duplicate samples from nine ECHO sites, 76 analytes had ≤25% median relative percent differences (RPDs). In addition, 63 of 84 analytes (75%) with at least two valid duplicate pairs had intraclass correlation coefficients above 0.75.

Generally, chemicals with CVs >20% or RPDs >25% are those that were found at ultra-trace levels, i.e., close to the LOD. A small deviation at such levels close to instrument detection limit can result in high CVs or RPDs. Instrument-derived values, including those with negative values below the LOD, are prone to even higher uncertainties. Therefore, we modeled analytes with detection frequencies of 5–70% as binary variables and did not model analytes with detection frequencies <5%.

Values below limits of detection

When possible, the laboratory provided instrument values for concentrations below the LOD. To facilitate log transformation and use all available information on the rank-ordering of values, we used a tiered approach for values <LOD. (1) If no instrument value was reported, we used the value of LOD/sqrt(2).[4] (2) If the instrument value was

© 2026 Buckley JP et al. *JAMA Network Open.*

negative or below 0.01, we replaced it with 0.01. (3) If the instrument value was at or above 0.01, we used the reported instrument value.

© 2026 Buckley JP et al. *JAMA Network Open.*

eTable 16. Recovery Table for NIST Quality Control Samples

Chemical Class	Analyte	Pool	Target value	LOD	Total N	Valid N	% Valid	Mean	CV (%)	Mean Recovery (%)
Fungicides and Herbicides	24D (ng/mL)	SRM3672	NA	0.06	82	69	84	0.245	37	NC
		SRM3673	NA	0.06	82	68	83	0.204	39	NC
	METB (ng/mL)	SRM3672	NA	0.84	82	3	4	1.99	9	NC
		SRM3673	NA	0.84	82	1	1	1.56	NC	NC
	T245 (ng/mL)	SRM3672	NA	0.044	82	4	5	0.0674	4	NC
		SRM3673	NA	0.044	82	2	2	0.0568	32	NC
	ATZ (ng/mL)	SRM3672	NA	0.069	82	11	13	0.111	30	NC
		SRM3673	NA	0.069	82	2	2	0.0807	16	NC
	PRPCN (ng/mL)	SRM3672	NA	0.34	82	2	2	0.436	28	NC
		SRM3673	NA	0.34	82	1	1	0.356	NC	NC
	CYP (ng/mL)	SRM3672	NA	0.034	82	0	0	NC	NC	NC
		SRM3673	NA	0.034	82	2	2	0.0585	26	NC
	PYRM (ng/mL)	SRM3672	NA	0.075	82	1	1	0.0998	NC	NC
		SRM3673	NA	0.075	82	0	0	NC	NC	NC
	TETZ (ng/mL)	SRM3672	NA	0.26	82	0	0	NC	NC	NC
		SRM3673	NA	0.26	82	0	0	NC	NC	NC
	TBZ (ng/mL)	SRM3672	NA	0.089	82	0	0	NC	NC	NC
		SRM3673	NA	0.089	82	0	0	NC	NC	NC
	AZO (ng/mL)	SRM3672	NA	0.092	82	1	1	0.0957	NC	NC
		SRM3673	NA	0.092	82	0	0	NC	NC	NC
	MET (ng/mL)	SRM3672	NA	0.09	82	0	0	NC	NC	NC
		SRM3673	NA	0.09	82	0	0	NC	NC	NC
Insecticides: Synthetic Pyrethroids	PBA (ng/mL)	SRM3672	NA	0.028	82	82	100	0.471	13	NC
		SRM3673	NA	0.028	82	82	100	0.500	12	NC
	TDCCA (ng/mL)	SRM3672	NA	0.037	82	81	99	0.608	16	NC
		SRM3673	NA	0.037	82	81	99	0.649	15	NC
	CDCCA (ng/mL)	SRM3672	NA	0.20	82	19	23	0.476	17	NC
		SRM3673	NA	0.20	82	28	34	0.455	16	NC
	FPBA (ng/mL)	SRM3672	NA	0.034	82	0	0	NC	NC	NC
		SRM3673	NA	0.034	82	0	0	NC	NC	NC
Insecticides: Neonicotinoid Insecticides	NDMA (ng/mL)	SRM3672	NA	0.051	82	54	66	0.113	25	NC
		SRM3673	NA	0.051	82	55	67	0.137	33	NC
	THX (ng/mL)	SRM3672	NA	0.048	82	17	21	0.0721	11	NC

© 2026 Buckley JP et al. *JAMA Network Open.*

Chemical Class	Analyte	Pool	Target value	LOD	Total N	Valid N	% Valid	Mean	CV (%)	Mean Recovery (%)
		SRM3673	NA	0.048	82	9	11	0.0720	11	NC
	IMI (ng/mL)	SRM3672	NA	0.073	82	14	17	0.214	13	NC
		SRM3673	NA	0.073	82	16	20	0.137	11	NC
	NDMT (ng/mL)	SRM3672	NA	0.29	82	9	11	6.94	11	NC
		SRM3673	NA	0.29	82	11	13	0.588	13	NC
	ACE (ng/mL)	SRM3672	NA	0.039	82	22	27	0.0645	21	NC
		SRM3673	NA	0.039	82	11	13	0.0617	18	NC
	CLO (ng/mL)	SRM3672	NA	0.16	82	3	4	0.403	5	NC
		SRM3673	NA	0.16	82	4	5	0.255	27	NC
	TA (ng/mL)	SRM3672	NA	0.11	82	7	9	0.930	14	NC
		SRM3673	NA	0.11	82	10	12	0.178	13	NC
	IMZ (ng/mL)	SRM3672	NA	0.21	82	7	9	0.278	13	NC
		SRM3673	NA	0.21	82	10	12	0.667	12	NC
	NIT (ng/mL)	SRM3672	NA	0.056	82	6	7	0.143	10	NC
		SRM3673	NA	0.056	82	12	15	0.0947	10	NC
	DINF (ng/mL)	SRM3672	NA	0.16	82	7	9	0.203	14	NC
		SRM3673	NA	0.16	82	11	13	0.345	10	NC
	CINA6 (ng/mL)	SRM3672	NA	0.045	82	5	6	0.0609	12	NC
		SRM3673	NA	0.045	82	2	2	0.0940	11	NC
	FLNC (ng/mL)	SRM3672	NA	0.42	82	2	2	1.83	5	NC
		SRM3673	NA	0.42	82	1	1	5.62	NC	NC
	THI (ng/mL)	SRM3672	NA	0.062	82	3	4	0.110	4	NC
		SRM3673	NA	0.062	82	1	1	0.197	NC	NC
Insecticides: Other Insecticides	TCP (ng/mL)	SRM3672	NA	0.039	82	82	100	0.848	15	NC
		SRM3673	NA	0.039	82	82	100	0.637	11	NC
	PNP (ng/mL)	SRM3672	NA	0.19	82	48	59	0.527	37	NC
		SRM3673	NA	0.19	82	30	37	0.365	32	NC
	SUF (ng/mL)	SRM3672	NA	0.041	82	0	0	NC	NC	NC
		SRM3673	NA	0.041	82	0	0	NC	NC	NC
Halogenated Phenols	PCP (ng/mL)	SRM3672	NA	0.046	82	76	93	0.120	35	NC
		SRM3673	NA	0.046	82	77	94	0.132	31	NC
	TECP2346 (ng/mL)	SRM3672	NA	0.25	82	2	2	0.525	22	NC
		SRM3673	NA	0.25	82	0	0	NC	NC	NC
	TCP245/TCP246 (ng/mL)	SRM3672	NA	0.39	82	7	9	0.798	19	NC
		SRM3673	NA	0.39	82	10	12	0.596	17	NC
	TECP2356 (ng/mL)	SRM3672	NA	0.21	82	5	6	0.245	12	NC

© 2026 Buckley JP et al. *JAMA Network Open.*

Chemical Class	Analyte	Pool	Target value	LOD	Total N	Valid N	% Valid	Mean	CV (%)	Mean Recovery (%)
		SRM3673	NA	0.21	82	1	1	0.220	NC	NC
	TECP2345 (ng/mL)	SRM3672	NA	0.34	82	0	0	NC	NC	NC
		SRM3673	NA	0.34	82	0	0	NC	NC	NC
Organophosphate Esters	BDCPP (ng/mL)	SRM3672	NA	0.076	82	72	88	0.222	33	NC
		SRM3673	NA	0.076	82	77	94	0.551	37	NC
	DPHP (ng/mL)	SRM3672	NA	0.25	82	79	96	0.889	18	NC
		SRM3673	NA	0.25	82	71	87	0.450	15	NC
	TEP (ng/mL)	SRM3672	NA	0.12	82	66	80	0.255	33	NC
		SRM3673	NA	0.12	82	49	60	0.224	36	NC
	TPHP (ng/mL)	SRM3672	NA	0.11	82	20	24	0.181	18	NC
		SRM3673	NA	0.11	82	22	27	0.261	25	NC
	DBUP/DIBP (ng/mL)	SRM3672	NA	0.15	82	27	33	0.200	17	NC
		SRM3673	NA	0.15	82	2	2	0.501	12	NC
	TBOEP (ng/mL)	SRM3672	NA	0.20	82	8	10	0.234	13	NC
		SRM3673	NA	0.20	82	15	18	0.397	15	NC
	TNBP/TIBP (ng/mL)	SRM3672	NA	0.25	82	3	4	0.584	24	NC
		SRM3673	NA	0.25	82	7	9	0.394	13	NC
	DoCP (ng/mL)	SRM3672	NA	0.28	82	1	1	1.13	NC	NC
		SRM3673	NA	0.28	82	0	0	NC	NC	NC
	TCEP (ng/mL)	SRM3672	NA	0.11	82	1	1	0.354	NC	NC
		SRM3673	NA	0.11	82	4	5	0.133	20	NC
	TPP (ng/mL)	SRM3672	NA	0.31	82	0	0	NC	NC	NC
		SRM3673	NA	0.31	82	0	0	NC	NC	NC
Benzophenones	BP1 (ng/mL)	SRM3672	NA	0.044	82	82	100	50.3	15	NC
		SRM3673	NA	0.044	82	82	100	57.6	15	NC
	BP3 (ng/mL)	SRM3672	187	0.053	82	82	100	212	15	114
		SRM3673	279	0.053	82	82	100	303	18	108
	BP8 (ng/mL)	SRM3672	NA	0.14	82	80	98	0.306	36	NC
		SRM3673	NA	0.14	82	59	72	0.211	28	NC
	OH4BP (ng/mL)	SRM3672	NA	0.17	82	82	100	0.461	36	NC
		SRM3673	NA	0.17	82	76	93	0.435	35	NC
	BP6 (ng/mL)	SRM3672	NA	0.063	82	30	37	0.323	27	NC
		SRM3673	NA	0.063	82	26	32	0.409	31	NC
	BP2 (ng/mL)	SRM3672	NA	0.22	82	0	0	NC	NC	NC
		SRM3673	NA	0.22	82	0	0	NC	NC	NC
Bisphenols	BPS (ng/mL)	SRM3672	NA	0.048	82	80	98	0.214	31	NC

© 2026 Buckley JP et al. *JAMA Network Open.*

Chemical Class	Analyte	Pool	Target value	LOD	Total N	Valid N	% Valid	Mean	CV (%)	Mean Recovery (%)
		SRM3673	NA	0.048	82	76	93	0.143	33	NC
	BPF (ng/mL)	SRM3672	NA	0.052	82	64	78	1.11	26	NC
		SRM3673	NA	0.052	82	67	82	1.02	37	NC
	BPA (ng/mL)	SRM3672	3.11	0.056	82	77	94	3.10	10	100
		SRM3673	2.00	0.056	82	66	80	1.88	9	94
	BADGE (ng/mL)	SRM3672	NA	0.18	82	6	7	1.44	10	NC
		SRM3673	NA	0.18	82	12	15	0.250	10	NC
	BPAP (ng/mL)	SRM3672	NA	0.21	82	2	2	0.276	6	NC
		SRM3673	NA	0.21	82	3	4	0.269	22	NC
	BPP (ng/mL)	SRM3672	NA	0.055	82	4	5	0.0713	16	NC
		SRM3673	NA	0.055	82	5	6	0.0647	7	NC
	BADGE2H2O (ng/mL)	SRM3672	NA	0.63	82	0	0	NC	NC	NC
		SRM3673	NA	0.63	82	2	2	1.45	47	NC
	BPB (ng/mL)	SRM3672	NA	0.35	82	2	2	0.390	4	NC
		SRM3673	NA	0.35	82	2	2	0.661	2	NC
	BPZ (ng/mL)	SRM3672	NA	0.083	82	2	2	0.113	24	NC
		SRM3673	NA	0.083	82	2	2	0.0988	17	NC
	BADGEH2O (ng/mL)	SRM3672	NA	0.68	82	9	11	1.68	13	NC
		SRM3673	NA	0.68	82	6	7	2.50	14	NC
	BPAF (ng/mL)	SRM3672	NA	0.036	82	0	0	NC	NC	NC
		SRM3673	NA	0.036	82	0	0	NC	NC	NC
	TBBPA3355 (ng/mL)	SRM3672	NA	0.083	82	0	0	NC	NC	NC
		SRM3673	NA	0.083	82	0	0	NC	NC	NC
	TECBPA2266 (ng/mL)	SRM3672	NA	0.26	82	0	0	NC	NC	NC
		SRM3673	NA	0.26	82	0	0	NC	NC	NC
	TrCBPA335 (ng/mL)	SRM3672	NA	0.34	82	0	0	NC	NC	NC
		SRM3673	NA	0.34	82	0	0	NC	NC	NC
Parabens	MEPB (ng/mL)	SRM3672	115	0.027	82	82	100	102	15	89
		SRM3673	81.0	0.027	82	82	100	78.7	15	97
	PRPB (ng/mL)	SRM3672	17.9	0.028	82	82	100	18.0	11	100
		SRM3673	22.0	0.028	82	82	100	21.8	10	99
	ETPB (ng/mL)	SRM3672	8.27	0.072	82	82	100	7.05	13	85
		SRM3673	10.5	0.072	82	82	100	8.87	12	84
	BUPB (ng/mL)	SRM3672	11.3	0.10	82	82	100	10.7	12	95
		SRM3673	1.13	0.10	82	82	100	1.07	14	95

© 2026 Buckley JP et al. *JAMA Network Open.*

Chemical Class	Analyte	Pool	Target value	LOD	Total N	Valid N	% Valid	Mean	CV (%)	Mean Recovery (%)
	BZPB (ng/mL)	SRM3672	NA	0.052	82	67	82	0.0690	16	NC
		SRM3673	NA	0.052	82	0	0	NC	NC	NC
	HEPB (ng/mL)	SRM3672	NA	0.17	82	0	0	NC	NC	NC
		SRM3673	NA	0.17	82	0	0	NC	NC	NC
Antimicrobials	TCC (ng/mL)	SRM3672	NA	0.045	82	31	38	0.0691	36	NC
		SRM3673	NA	0.045	82	82	100	2.60	36	NC
	TCS (ng/mL)	SRM3672	18.0	0.068	82	82	100	15.6	11	86
		SRM3673	6.39	0.068	82	82	100	5.65	11	88
Phthalates & Alternatives	MEHHP (ng/mL)	SRM3672	25.3	0.066	82	82	100	27.8	10	110
		SRM3673	22.7	0.066	82	82	100	24.7	10	109
	MEOHP (ng/mL)	SRM3672	15.2	0.054	82	82	100	14.4	10	95
		SRM3673	12.4	0.054	82	82	100	12.0	9	97
	MECPP (ng/mL)	SRM3672	35.9	0.35	82	82	100	30.8	14	86
		SRM3673	30.7	0.35	82	82	100	26.6	13	87
	MCMHP (ng/mL)	SRM3672	NA	0.18	82	82	100	7.27	18	NC
		SRM3673	NA	0.18	82	82	100	5.70	18	NC
	MEHP (ng/mL)	SRM3672	4.21	0.094	82	82	100	4.35	18	103
		SRM3673	4.42	0.094	82	82	100	4.62	18	105
	MCIOP (ng/mL)	SRM3672	21.7	0.046	82	82	100	22.2	11	102
		SRM3673	10.7	0.046	82	82	100	11.0	11	103
	MHINP (ng/mL)	SRM3672	NA	0.12	82	82	100	2.95	18	NC
		SRM3673	NA	0.12	82	82	100	1.38	17	NC
	MPCHP (ng/mL)	SRM3672	NA	0.050	82	82	100	0.818	11	NC
		SRM3673	NA	0.050	82	82	100	0.635	11	NC
	MPOHP (ng/mL)	SRM3672	NA	0.044	82	81	99	0.251	29	NC
		SRM3673	NA	0.044	82	57	70	0.0776	33	NC
	MPHHP (ng/mL)	SRM3672	NA	0.063	82	5	6	0.492	15	NC
		SRM3673	NA	0.063	82	1	1	0.616	NC	NC
	MCINP (ng/mL)	SRM3672	1.96	0.060	82	82	100	1.88	14	96
		SRM3673	1.52	0.060	82	82	100	1.43	13	94
	MHIDP (ng/mL)	SRM3672	NA	0.16	82	3	4	0.834	2	NC
		SRM3673	NA	0.16	82	0	0	NC	NC	NC
	MIDP (ng/mL)	SRM3672	NA	0.060	82	2	2	0.526	11	NC
		SRM3673	NA	0.060	82	5	6	0.229	18	NC
	MBZP (ng/mL)	SRM3672	8.53	0.084	82	82	100	9.33	12	109

© 2026 Buckley JP et al. *JAMA Network Open.*

Chemical Class	Analyte	Pool	Target value	LOD	Total N	Valid N	% Valid	Mean	CV (%)	Mean Recovery (%)
		SRM3673	5.80	0.084	82	82	100	6.30	11	109
	MCPP (ng/mL)	SRM3672	3.05	0.058	82	82	100	2.76	11	90
		SRM3673	1.95	0.058	82	82	100	1.74	9	89
	MCHPP (ng/mL)	SRM3672	NA	0.23	82	81	99	9.10	16	NC
		SRM3673	NA	0.23	82	80	98	4.00	17	NC
	MOP (ng/mL)	SRM3672	NA	0.066	82	0	0	NC	NC	NC
		SRM3673	NA	0.066	82	1	1	0.198	NC	NC
	MHPP (ng/mL)	SRM3672	NA	0.051	82	79	96	1.20	12	NC
		SRM3673	NA	0.051	82	76	93	0.670	15	NC
	MHXP (ng/mL)	SRM3672	NA	0.039	82	78	95	0.135	15	NC
		SRM3673	NA	0.039	82	81	99	0.236	16	NC
	MPRP (ng/mL)	SRM3672	NA	0.11	82	0	0	NC	NC	NC
		SRM3673	NA	0.11	82	2	2	0.190	13	NC
	MPEP (ng/mL)	SRM3672	NA	0.042	82	2	2	0.0488	10	NC
		SRM3673	NA	0.042	82	1	1	0.0478	NC	NC
	METP (ng/mL)	SRM3672	NA	0.082	82	16	20	0.131	12	NC
		SRM3673	NA	0.082	82	2	2	0.0953	15	NC
	MTBTP (ng/mL)	SRM3672	NA	0.075	82	0	0	NC	NC	NC
		SRM3673	NA	0.075	82	0	0	NC	NC	NC
	MBZTP (ng/mL)	SRM3672	NA	0.088	82	0	0	NC	NC	NC
		SRM3673	NA	0.088	82	0	0	NC	NC	NC
	MEP (ng/mL)	SRM3672	96.3	0.075	82	82	100	115	14	119
		SRM3673	81.7	0.075	82	82	100	94.6	11	116
	MIBP/MNBP (ng/mL)	SRM3672	NA	0.15	82	82	100	18.4	12	NC
		SRM3673	NA	0.15	82	82	100	17.7	10	NC
	MMP (ng/mL)	SRM3672	NA	0.32	82	27	33	1.30	21	NC
		SRM3673	NA	0.32	82	26	32	1.28	28	NC
	MIPP (ng/mL)	SRM3672	NA	0.099	82	8	10	0.137	12	NC
		SRM3673	NA	0.099	82	5	6	0.116	6	NC
	MHNCH (ng/mL)	SRM3672	NA	0.037	82	61	74	0.0687	20	NC
		SRM3673	NA	0.037	82	3	4	0.0420	15	NC
	MONCH (ng/mL)	SRM3672	NA	0.045	82	44	54	0.0584	17	NC
		SRM3673	NA	0.045	82	8	10	0.0513	10	NC
	MCOCH (ng/mL)	SRM3672	NA	0.056	82	5	6	0.0792	13	NC
		SRM3673	NA	0.056	82	1	1	0.0648	NC	NC
	PA (ng/mL)	SRM3672	NA	5.0	82	1	1	5.74	NC	NC

© 2026 Buckley JP et al. *JAMA Network Open.*

Chemical Class	Analyte	Pool	Target value	LOD	Total N	Valid N	% Valid	Mean	CV (%)	Mean Recovery (%)
		SRM3673	NA	5.0	82	0	0	NC	NC	NC
Polycyclic Aromatic Hydrocarbons	NAP2 (ng/L)	SRM3672	8730	54	82	78	95	8460	29	97
		SRM3673	1350	54	82	76	93	1320	22	98
	NAP1 (ng/L)	SRM3672	34400	110	82	78	95	38300	29	111
		SRM3673	211000	110	82	82	100	185000	24	88
	PHEN1/PHEN9 (ng/L)	SRM3672	NA	190	82	51	62	934	33	NC
		SRM3673	NA	190	82	4	5	221	4	NC
	PYR1 (ng/L)	SRM3672	173	58	82	79	96	178	18	103
		SRM3673	30.5	58	82	2	2	81.2	13	266
	PHEN2/PHEN3 (ng/L)	SRM3672	NA	110	82	77	94	224	18	NC
		SRM3673	NA	110	82	1	1	123	NC	NC
	FLUO2/3/9 (ng/L)	SRM3672	NA	350	82	82	100	1750	22	NC
		SRM3673	NA	350	82	5	6	396	14	NC
	PHEN4 (ng/L)	SRM3672	48.9	150	82	2	2	341	13	698
		SRM3673	10.4	150	82	0	0	NC	NC	NC

Abbreviations: CV, coefficient of variation; LOD, limit of detection; NA, not applicable; NC, not calculated.

© 2026 Buckley JP et al. *JAMA Network Open.*

eTable 17. Summary of Results for Quality Control Spikes

Chemical Class	Analyte	LOD	Total N	Valid N	% Valid	Mean	CV (%)
Fungicides and Herbicides	24D (ng/mL)	0.060	164	163	99	10.3	8
	METB (ng/mL)	0.84	164	162	99	10.8	30
	T245 (ng/mL)	0.044	164	164	100	10.2	7
	ATZ (ng/mL)	0.069	164	164	100	10.9	16
	PRPCN (ng/mL)	0.34	164	163	99	10.8	21
	CYP (ng/mL)	0.034	164	163	99	9.77	15
	PYRM (ng/mL)	0.075	164	163	99	10.7	18
	TETZ (ng/mL)	0.26	164	163	99	9.94	25
	TBZ (ng/mL)	0.089	164	163	99	9.94	10
	AZO (ng/mL)	0.092	164	163	99	10.2	19
	MET (ng/mL)	0.090	164	164	100	10.0	10
Insecticides: Synthetic Pyrethroids	PBA (ng/mL)	0.028	164	164	100	10.1	8
	TDCCA (ng/mL)	0.037	164	164	100	12.7	9
	CDCCA (ng/mL)	0.20	164	164	100	11.8	13
	FPBA (ng/mL)	0.034	164	164	100	10.1	6
Insecticides: Neonicotinoid Insecticides	NDMA (ng/mL)	0.051	164	164	100	10.5	11
	THX (ng/mL)	0.048	164	163	99	9.43	12
	IMI (ng/mL)	0.073	164	163	99	10.1	13
	NDMT (ng/mL)	0.29	164	163	99	13.2	28
	ACE (ng/mL)	0.039	164	164	100	10.2	11
	CLO (ng/mL)	0.16	164	163	99	10.4	24
	TA (ng/mL)	0.11	164	163	99	9.54	11
	IMZ (ng/mL)	0.21	164	163	99	10.1	20
	NIT (ng/mL)	0.056	164	163	99	9.87	14

© 2026 Buckley JP et al. *JAMA Network Open.*

Chemical Class	Analyte	LOD	Total N	Valid N	% Valid	Mean	CV (%)
	DINF (ng/mL)	0.16	164	159	97	9.79	20
	CINA6 (ng/mL)	0.045	164	164	100	10.1	10
	FLNC (ng/mL)	0.42	164	163	99	10.6	27
	THI (ng/mL)	0.062	164	164	100	10.2	13
Insecticides: Other Insecticides	TCP (ng/mL)	0.039	164	164	100	10.8	7
	PNP (ng/mL)	0.19	164	157	96	10.4	18
	SUF (ng/mL)	0.041	164	163	99	10.4	9
Halogenated Phenols	PCP (ng/mL)	0.046	164	163	99	10.9	14
	TECP2346 (ng/mL)	0.25	164	152	93	10.3	24
	TCP245/TCP246 (ng/mL)	0.39	164	163	99	15.7	24
	TECP2356 (ng/mL)	0.21	164	152	93	10.3	28
	TECP2345 (ng/mL)	0.34	164	150	91	11.5	28
Organophosphate Esters	BDCPP (ng/mL)	0.076	164	163	99	9.90	14
	DPHP (ng/mL)	0.25	164	164	100	10.6	16
	TEP (ng/mL)	0.12	164	163	99	11.4	14
	TPHP (ng/mL)	0.11	164	163	99	9.82	18
	DBUP/DIBP (ng/mL)	0.15	164	164	100	21.3	10
	TBOEP (ng/mL)	0.20	164	163	99	11.2	17
	TNBP/TIBP (ng/mL)	0.25	164	163	99	18.7	14
	DoCP (ng/mL)	0.28	164	163	99	9.38	29
	TCEP (ng/mL)	0.11	164	163	99	10.3	26
	TPP (ng/mL)	0.31	164	164	100	10.2	13
Benzophenones	BP1 (ng/mL)	0.044	164	163	99	10.3	10
	BP3 (ng/mL)	0.053	164	162	99	10.8	19
	BP8 (ng/mL)	0.14	164	163	99	11.0	27
	OH4BP (ng/mL)	0.17	164	163	99	12.1	28
	BP6 (ng/mL)	0.063	164	162	99	11.7	19

© 2026 Buckley JP et al. *JAMA Network Open.*

Chemical Class	Analyte	LOD	Total N	Valid N	% Valid	Mean	CV (%)
	BP2 (ng/mL)	0.22	164	163	99	8.52	28
Bisphenols	BPS (ng/mL)	0.048	164	163	99	10.5	10
	BPF (ng/mL)	0.052	164	163	99	12.2	46
	BPA (ng/mL)	0.056	164	163	99	26.9	8
	BADGE (ng/mL)	0.18	164	163	99	10.4	28
	BPAP (ng/mL)	0.21	164	163	99	10.4	25
	BPP (ng/mL)	0.055	164	163	99	9.84	12
	BADGE2H2O (ng/mL)	0.63	164	163	99	11.5	29
	BPB (ng/mL)	0.35	164	163	99	11.9	30
	BPZ (ng/mL)	0.083	164	163	99	9.75	11
	BADGEH2O (ng/mL)	0.68	164	162	99	12.0	29
	BPAF (ng/mL)	0.036	164	163	99	10.6	12
	TBBPA3355 (ng/mL)	0.083	164	163	99	9.85	17
	TECBPA2266 (ng/mL)	0.26	164	163	99	13.0	23
	TrCBPA335 (ng/mL)	0.34	164	163	99	13.6	25
Parabens	MEPB (ng/mL)	0.027	164	163	99	10.9	9
	PRPB (ng/mL)	0.028	164	163	99	10.4	11
	ETPB (ng/mL)	0.072	164	163	99	10.5	12
	BUPB (ng/mL)	0.10	164	163	99	9.37	15
	BZPB (ng/mL)	0.052	164	163	99	9.44	14
	HEPB (ng/mL)	0.17	164	163	99	9.02	14
Antimicrobials	TCS (ng/mL)	0.068	164	163	99	9.74	13
	TCC (ng/mL)	0.045	164	163	99	10.3	10
Phthalates & Alternatives	MEHHP (ng/mL)	0.066	164	164	100	10.4	10
	MEOHP (ng/mL)	0.054	164	164	100	9.82	7
	MECPP (ng/mL)	0.35	164	164	100	9.21	14
	MCMHP (ng/mL)	0.18	164	162	99	9.61	17
	MEHP (ng/mL)	0.094	164	164	100	18.0	15
	MCIOP (ng/mL)	0.046	164	164	100	9.76	8

© 2026 Buckley JP et al. *JAMA Network Open.*

Chemical Class	Analyte	LOD	Total N	Valid N	% Valid	Mean	CV (%)
	MHNCH (ng/mL)	0.037	164	164	100	9.93	6
	MPCHP (ng/mL)	0.050	164	164	100	9.80	7
	MPOHP (ng/mL)	0.044	164	164	100	9.80	6
	MPHHP (ng/mL)	0.063	164	164	100	9.72	10
	MCINP (ng/mL)	0.060	164	164	100	9.72	7
	MHIDP (ng/mL)	0.16	164	164	100	10.8	14
	MIDP (ng/mL)	0.060	164	164	100	9.84	10
	MBZP (ng/mL)	0.084	164	164	100	9.90	12
	MCPP (ng/mL)	0.058	164	164	100	8.43	11
	MCHPP (ng/mL)	0.23	164	164	100	14.9	11
	MOP (ng/mL)	0.066	164	164	100	9.69	10
	MHPP (ng/mL)	0.051	164	164	100	9.91	10
	MHXP (ng/mL)	0.039	164	164	100	9.81	8
	MPRP (ng/mL)	0.11	164	164	100	10.7	13
	MPEP (ng/mL)	0.042	164	164	100	10.1	9
	METP (ng/mL)	0.082	164	164	100	9.76	15
	MTBTP (ng/mL)	0.075	164	164	100	9.70	15
	MBZTP (ng/mL)	0.088	164	160	98	8.69	17
	MEP (ng/mL)	0.075	164	164	100	9.96	8
	MNBP/MIBP (ng/mL)	0.15	164	164	100	21.1	9
	MMP (ng/mL)	0.32	164	164	100	12.3	13
	MIPP (ng/mL)	0.099	164	164	100	10.1	11
	MHINP (ng/mL)	0.12	164	164	100	15.2	12
	MONCH (ng/mL)	0.045	164	164	100	10.0	7
	MCOCH (ng/mL)	0.056	164	164	100	9.67	12
	PA (ng/mL)	5.0	164	153	93	14.8	20
Polycyclic Aromatic Hydrocarbons	NAP2 (ng/L)	54	164	163	99	10300	11
	NAP1 (ng/L)	110	164	161	98	10200	29
	PHEN1/PHEN9 (ng/L)	190	164	163	99	20400	29

© 2026 Buckley JP et al. *JAMA Network Open.*

Chemical Class	Analyte	LOD	Total N	Valid N	% Valid	Mean	CV (%)
	PYR1 (ng/L)	58	164	151	92	10500	14
	PHEN2/PHEN3 (ng/L)	110	164	163	99	20600	9
	FLUO2/3/9 (ng/L)	350	164	163	99	30300	19
	PHEN4 (ng/L)	150	164	163	99	9700	29

Abbreviations: CV, coefficient of variation; LOD, limit of detection.

© 2026 Buckley JP et al. *JAMA Network Open.*

eTable 18. Summary of Results of HHEAR Quality Control (QC) Pools

Chemical Class	Analyte	Pool	LOD	Total N	Valid N	% Valid	Mean	CV (%)
Fungicides and Herbicides	24D (ng/mL)	H-A	0.06	246	202	82	0.267	29
		H-B	0.06	246	210	85	0.347	21
	METB (ng/mL)	H-A	0.039	246	48	20	0.0755	34
		H-B	0.039	246	85	35	0.118	34
	T245 (ng/mL)	H-A	0.069	246	10	4	0.0987	21
		H-B	0.069	246	20	8	0.108	15
	ATZ (ng/mL)	H-A	0.092	246	0	0	NC	NC
		H-B	0.092	246	0	0	NC	NC
	PRPCN (ng/mL)	H-A	0.18	246	7	3	0.469	12
		H-B	0.18	246	8	3	0.883	23
	CYP (ng/mL)	H-A	0.63	246	2	1	1.01	7
		H-B	0.63	246	5	2	1.08	31
	PYRM (ng/mL)	H-A	0.68	246	4	2	2.98	19
		H-B	0.68	246	6	2	3.53	40
	TETZ (ng/mL)	H-A	0.076	246	224	91	1.53	20
		H-B	0.076	246	231	94	1.82	26
	TBZ (ng/mL)	H-A	0.044	246	246	100	26.7	12
		H-B	0.044	246	246	100	37.3	15
	AZO (ng/mL)	H-A	0.22	246	0	0	NC	NC
		H-B	0.22	246	0	0	NC	NC
	MET (ng/mL)	H-A	0.053	246	243	99	362	23
		H-B	0.053	246	245	100	167	23
Insecticides: Synthetic Pyrethroids	PBA (ng/mL)	H-A	0.063	246	75	30	0.412	35
		H-B	0.063	246	82	33	0.888	29
	TDCCA (ng/mL)	H-A	0.14	246	243	99	0.430	31
		H-B	0.14	246	214	87	0.333	36
	CDCCA (ng/mL)	H-A	0.056	246	20	8	0.101	23
		H-B	0.056	246	218	89	2.33	22
	FPBA (ng/mL)	H-A	0.036	246	0	0	NC	NC
		H-B	0.036	246	2	1	0.0409	8
Insecticides: Neonicotinoid Insecticides	NDMA (ng/mL)	H-A	0.21	246	3	1	0.244	4
		H-B	0.21	246	7	3	0.284	25
	THX (ng/mL)	H-A	0.35	246	3	1	0.442	14

© 2026 Buckley JP et al. *JAMA Network Open.*

Chemical Class	Analyte	Pool	LOD	Total N	Valid N	% Valid	Mean	CV (%)
		H-B	0.35	246	13	5	0.588	21
	IMI (ng/mL)	H-A	0.052	246	189	77	0.548	36
		H-B	0.052	246	200	81	1.04	36
	NDMT (ng/mL)	H-A	0.055	246	13	5	0.0828	19
		H-B	0.055	246	13	5	0.152	14
	ACE (ng/mL)	H-A	0.048	246	169	69	0.232	29
		H-B	0.048	246	246	100	1.91	17
	CLO (ng/mL)	H-A	0.083	246	3	1	0.103	9
		H-B	0.083	246	4	2	0.147	34
	TA (ng/mL)	H-A	0.10	246	246	100	2.20	16
		H-B	0.10	246	27	11	0.129	20
	IMZ (ng/mL)	H-A	0.052	246	212	86	0.0764	17
		H-B	0.052	246	48	20	0.0626	14
	NIT (ng/mL)	H-A	0.20	246	12	5	0.564	7
		H-B	0.20	246	13	5	1.66	19
	DINF (ng/mL)	H-A	0.045	246	3	1	0.0544	17
		H-B	0.045	246	7	3	0.0897	15
	CINA6 (ng/mL)	H-A	0.16	246	15	6	0.239	17
		H-B	0.16	246	24	10	0.362	26
	FLNC (ng/mL)	H-A	0.034	246	1	0	0.0384	NC
		H-B	0.034	246	2	1	0.0405	21
	THI (ng/mL)	H-A	0.15	246	6	2	0.279	10
		H-B	0.15	246	29	12	0.192	19
Insecticides: Other Insecticides	TCP (ng/mL)	H-A	0.16	246	216	88	1.09	25
		H-B	0.16	246	53	22	0.298	29
	PNP (ng/mL)	H-A	0.28	246	1	0	1.05	NC
		H-B	0.28	246	2	1	0.303	3
	SUF (ng/mL)	H-A	0.25	246	239	97	0.933	17
		H-B	0.25	246	240	98	3.14	17
Halogenated Phenols	PCP (ng/mL)	H-A	0.072	246	246	100	6.17	14
		H-B	0.072	246	246	100	5.55	14
	TECP2346 (ng/mL)	H-A	0.42	246	3	1	0.490	14
		H-B	0.42	246	22	9	0.635	27
	TCP245/TCP246 (ng/mL)	H-A	350	246	44	18	400	12
		H-B	350	246	246	100	3840	18
	TECP2356 (ng/mL)	H-A	0.034	246	0	0	NC	NC

© 2026 Buckley JP et al. *JAMA Network Open.*

Chemical Class	Analyte	Pool	LOD	Total N	Valid N	% Valid	Mean	CV (%)
		H-B	0.034	246	30	12	0.483	12
	TECP2345 (ng/mL)	H-A	0.17	246	0	0	NC	NC
		H-B	0.17	246	0	0	NC	NC
Organophosphate Esters	BDCPP (ng/mL)	H-A	0.073	246	56	23	0.130	29
		H-B	0.073	246	45	18	1.17	25
	DPHP (ng/mL)	H-A	0.21	246	11	4	0.522	38
		H-B	0.21	246	28	11	1.03	29
	TEP (ng/mL)	H-A	0.084	246	246	100	14.1	13
		H-B	0.084	246	217	88	1.64	19
	TPHP (ng/mL)	H-A	0.088	246	0	0	NC	NC
		H-B	0.088	246	0	0	NC	NC
	DBUP/DIBP (ng/mL)	H-A	0.23	246	228	93	0.774	22
		H-B	0.23	246	235	96	0.799	20
	TBOEP (ng/mL)	H-A	0.060	246	246	100	0.555	20
		H-B	0.060	246	246	100	1.04	18
	TNBP/TIBP (ng/mL)	H-A	0.046	246	246	100	3.19	12
		H-B	0.046	246	246	100	6.28	12
	DoCP (ng/mL)	H-A	0.18	246	164	67	0.519	17
		H-B	0.18	246	238	97	1.43	18
	TCEP (ng/mL)	H-A	0.056	246	236	96	0.214	19
		H-B	0.056	246	238	97	0.461	22
	TPP (ng/mL)	H-A	0.058	246	246	100	3.47	14
		H-B	0.058	246	243	99	1.10	15
Benzophenones	BP1 (ng/mL)	H-A	0.35	246	246	100	2.25	15
		H-B	0.35	246	246	100	8.33	15
	BP3 (ng/mL)	H-A	0.066	246	246	100	3.00	14
		H-B	0.066	246	246	100	4.91	16
	BP8 (ng/mL)	H-A	0.094	246	11	4	0.272	55
		H-B	0.094	246	110	45	1.04	50
	OH4BP (ng/mL)	H-A	0.054	246	246	100	1.53	11
		H-B	0.054	246	246	100	1.92	15
	BP6 (ng/mL)	H-A	0.075	246	246	100	29.4	7
		H-B	0.075	246	246	100	86.9	9
	BP2 (ng/mL)	H-A	0.027	246	246	100	96.4	15
		H-B	0.027	246	246	100	58.9	17
Bisphenols	BPS (ng/mL)	H-A	0.09	246	0	0	NC	NC
		H-B	0.09	246	0	0	NC	NC

© 2026 Buckley JP et al. *JAMA Network Open.*

Chemical Class	Analyte	Pool	LOD	Total N	Valid N	% Valid	Mean	CV (%)
	BPF (ng/mL)	H-A	0.84	246	8	3	1.15	24
		H-B	0.84	246	6	2	1.30	19
	BPA (ng/mL)	H-A	0.082	246	2	1	0.0894	8
		H-B	0.082	246	213	87	0.426	17
	BADGE (ng/mL)	H-A	0.16	246	101	41	0.237	15
		H-B	0.16	246	244	99	0.483	15
	BPAP (ng/mL)	H-A	0.12	246	191	78	0.194	17
		H-B	0.12	246	193	78	0.239	16
	BPP (ng/mL)	H-A	0.037	246	246	100	0.507	11
		H-B	0.037	246	246	100	1.32	11
	BADGE2H2O (ng/mL)	H-A	0.051	246	147	60	0.0898	20
		H-B	0.051	246	103	42	0.0803	15
	BPB (ng/mL)	H-A	0.039	246	222	90	0.0855	15
		H-B	0.039	246	53	22	0.0512	16
	BPZ (ng/mL)	H-A	0.15	246	246	100	11.4	11
		H-B	0.15	246	246	100	11.0	14
	BADGEH2O (ng/mL)	H-A	0.060	246	6	2	0.215	6
		H-B	0.060	246	6	2	0.204	12
	BPAF (ng/mL)	H-A	0.099	246	246	100	0.912	16
		H-B	0.099	246	185	75	0.240	24
	TBBPA3355 (ng/mL)	H-A	0.32	246	153	62	1.30	24
		H-B	0.32	246	142	58	3.04	24
	TECBPA2266 (ng/mL)	H-A	0.045	246	246	100	0.202	14
		H-B	0.045	246	246	100	0.407	15
	TrCBPA335 (ng/mL)	H-A	0.066	246	0	0	NC	NC
		H-B	0.066	246	0	0	NC	NC
Parabens	MEPB (ng/mL)	H-A	0.050	246	246	100	0.241	12
		H-B	0.050	246	246	100	0.450	12
	PRPB (ng/mL)	H-A	0.042	246	1	0	0.0561	NC
		H-B	0.042	246	1	0	0.120	NC
	ETPB (ng/mL)	H-A	0.063	246	240	98	0.145	22
		H-B	0.063	246	243	99	0.526	14
	BUPB (ng/mL)	H-A	0.044	246	246	100	0.735	11
		H-B	0.044	246	246	100	1.52	15
	BZPB (ng/mL)	H-A	0.11	246	2	1	0.126	8
		H-B	0.11	246	2	1	0.116	6

© 2026 Buckley JP et al. *JAMA Network Open.*

Chemical Class	Analyte	Pool	LOD	Total N	Valid N	% Valid	Mean	CV (%)
	HEPB (ng/mL)	H-A	0.075	246	0	0	NC	NC
		H-B	0.075	246	2	1	0.0847	14
Antimicrobials	TCC (ng/mL)	H-A	110	246	203	83	1770	39
		H-B	110	246	222	90	17000	38
	TCS (ng/mL)	H-A	54	246	245	100	8280	21
		H-B	54	246	245	100	19200	31
Phthalates & Alternatives	MEHHP (ng/mL)	H-A	0.051	246	170	69	0.186	30
		H-B	0.051	246	178	72	0.265	35
	MEOHP (ng/mL)	H-A	0.29	246	8	3	1.55	22
		H-B	0.29	246	33	13	2.86	35
	MECPP (ng/mL)	H-A	0.056	246	11	4	0.0924	22
		H-B	0.056	246	25	10	2.87	31
	MCMHP (ng/mL)	H-A	0.17	246	157	64	0.354	32
		H-B	0.17	246	177	72	0.331	34
	MEHP (ng/mL)	H-A	5.0	246	1	0	6.06	NC
		H-B	5.0	246	6	2	13.1	14
	MCIOP (ng/mL)	H-A	0.028	246	246	100	0.350	11
		H-B	0.028	246	246	100	0.608	15
	MHINP (ng/mL)	H-A	0.046	246	236	96	0.575	18
		H-B	0.046	246	232	94	0.168	37
	MPCHP (ng/mL)	H-A	190	246	171	70	456	29
		H-B	190	246	212	86	1520	29
	MPOHP (ng/mL)	H-A	110	246	65	26	147	27
		H-B	110	246	246	100	686	21
	MPHHP (ng/mL)	H-A	150	246	8	3	211	21
		H-B	150	246	48	20	198	18
	MCINP (ng/mL)	H-A	0.19	246	67	27	0.302	29
		H-B	0.19	246	114	46	1.01	30
	MHIDP (ng/mL)	H-A	0.028	246	246	100	30.1	12
		H-B	0.028	246	246	100	12.9	12
	MIDP (ng/mL)	H-A	0.34	246	7	3	0.458	20
		H-B	0.34	246	5	2	0.461	15
	MBZP (ng/mL)	H-A	58	246	132	54	89.5	31
		H-B	58	246	246	100	478	26
	MCPP (ng/mL)	H-A	0.075	246	0	0	NC	NC
		H-B	0.075	246	8	3	0.152	28

© 2026 Buckley JP et al. *JAMA Network Open.*

Chemical Class	Analyte	Pool	LOD	Total N	Valid N	% Valid	Mean	CV (%)
	MCHPP (ng/mL)	H-A	0.041	246	23	9	0.0511	19
		H-B	0.041	246	246	100	0.0729	21
	MOP (ng/mL)	H-A	0.044	246	0	0	NC	NC
		H-B	0.044	246	0	0	NC	NC
	MHPP (ng/mL)	H-A	0.11	246	1	0	0.120	NC
		H-B	0.11	246	11	4	0.249	14
	MHXP (ng/mL)	H-A	0.083	246	3	1	0.159	5
		H-B	0.083	246	1	0	0.119	NC
	MPRP (ng/mL)	H-A	0.20	246	23	9	0.365	29
		H-B	0.20	246	34	14	0.364	33
	MPEP (ng/mL)	H-A	0.089	246	0	0	NC	NC
		H-B	0.089	246	2	1	0.108	6
	METP (ng/mL)	H-A	0.045	246	5	2	0.0611	11
		H-B	0.045	246	178	72	0.136	27
	MTBTP (ng/mL)	H-A	0.11	246	3	1	0.611	17
		H-B	0.11	246	0	0	NC	NC
	MBZTP (ng/mL)	H-A	0.039	246	246	100	0.456	13
		H-B	0.039	246	246	100	1.20	12
	MEP (ng/mL)	H-A	0.39	246	0	0	NC	NC
		H-B	0.39	246	13	5	0.467	13
	MIBP/MNBP (ng/mL)	H-A	0.068	246	234	95	0.360	33
		H-B	0.068	246	230	93	0.341	30
	MMP (ng/mL)	H-A	0.037	246	189	77	0.151	17
		H-B	0.037	246	127	52	0.121	15
	MIPP (ng/mL)	H-A	0.26	246	0	0	NC	NC
		H-B	0.26	246	0	0	NC	NC
	MHNCH (ng/mL)	H-A	0.34	246	3	1	0.589	21
		H-B	0.34	246	0	0	NC	NC
	MONCH (ng/mL)	H-A	0.25	246	29	12	0.303	14
		H-B	0.25	246	2	1	0.431	9
	MCOCH (ng/mL)	H-A	0.21	246	48	20	0.805	26
		H-B	0.21	246	11	4	0.255	20
	PA (ng/mL)	H-A	0.12	246	171	70	0.219	36
		H-B	0.12	246	210	85	0.281	38
Polycyclic Aromatic Hydrocarbons	NAP2 (ng/L)	H-A	0.26	246	0	0	NC	NC
		H-B	0.26	246	0	0	NC	NC

© 2026 Buckley JP et al. *JAMA Network Open.*

Chemical Class	Analyte	Pool	LOD	Total N	Valid N	% Valid	Mean	CV (%)
	NAP1 (ng/L)	H-A	0.062	246	1	0	0.0651	NC
		H-B	0.062	246	13	5	0.115	23
	PHEN1/PHEN9 (ng/L)	H-A	0.048	246	105	43	0.0828	31
		H-B	0.048	246	145	59	0.215	34
	PYR1 (ng/L)	H-A	0.25	246	15	6	0.603	39
		H-B	0.25	246	13	5	0.440	34
	PHEN2/PHEN3 (ng/L)	H-A	0.11	246	52	21	0.198	36
		H-B	0.11	246	77	31	0.259	36
	FLUO2/3/9 (ng/L)	H-A	0.31	246	0	0	NC	NC
		H-B	0.31	246	0	0	NC	NC
	PHEN4 (ng/L)	H-A	0.34	246	0	0	NC	NC
		H-B	0.34	246	0	0	NC	NC

Abbreviations: CV, coefficient of variation; H-A, HHEAR QC Pool A; H-B, HHEAR QC Pool B; LOD, limit of detection; NC, not calculated; QC, quality control.

© 2026 Buckley JP et al. *JAMA Network Open.*

eTable 19. Relative Percent Differences (RPDs) for Analytes Measured in Duplicate Samples from Nine ECHO Cohort Sites

Chemical Class	Analyte (acronym)	LOD (ng/mL)	Valid Pairs	RPD Median	RPD Max	ICC
Fungicides and Herbicides	24D	0.06	87	10.83	79.35	0.86
	METB	0.84	4	15.08	37.09	0.75
	T245	0.04	5	23.8	33.58	0.25
	ATZ	0.07	0	.	.	.
	PRPCN	0.34	0	.	.	.
	CYP	0.03	0	.	.	.
	PYRM	0.08	0	.	.	.
	TETZ	0.26	0	.	.	.
	TBZ	0.09	0	.	.	.
	AZO	0.09	0	.	.	.
	MET	0.09	0	.	.	.
Insecticides: Synthetic Pyrethroids	PBA	0.03	129	7.41	99.2	0.86
	TDCCA	0.04	96	13.88	96.59	0.87
	CDCCA	0.2	24	28.24	91.24	0.79
	FPBA	0.03	20	8.91	41.63	0.98
Insecticides: Neonicotinoid Insecticides	NDMA	0.05	65	11.6	55.52	0.95
	THX	0.05	18	10.18	36.33	0.9
	IMI	0.07	22	14.7	77.89	0.97
	NDMT	0.29	12	53.68	78.58	-0.04
	ACE	0.04	14	16.17	40	0.91
	CLO	0.16	7	10.4	53.95	0.85
	TA	0.11	0	.	.	.
	IMZ	0.21	5	29.55	77.21	-0.24
	NIT	0.06	3	66.01	78.45	-0.16
	DINF	0.16	5	6.69	51.01	0.96
	CINA6	0.05	3	29.29	38.56	0.77
	FLNC	0.42	2	28.65	31.65	0.19
	THI	0.06	4	24.09	36.89	0.57
Insecticides: Other Insecticides	TCP	0.04	133	7.42	33.89	0.95
	PNP	0.19	35	11.71	54.42	0.93
	SUF	0.04	7	2.44	8.53	0.97
Halogenated Phenols	PCP	0.05	83	11.79	58.35	0.98
	TECP2346	0.25	8	15.35	27.84	0.92
	TCP245/TCP246	0.39	3	16.31	22.4	0.66
	TECP2356	0.21	1	0.14	0.14	.
	TECP2345	0.34	0	.	.	.
Organophosphate Esters	BDCPP	0.08	107	9.67	80.1	0.79

© 2026 Buckley JP et al. *JAMA Network Open.*

Chemical Class	Analyte (acronym)	LOD (ng/mL)	Valid Pairs	RPD Median	RPD Max	ICC
	DPHP	0.25	100	18.66	75.23	0.89
	TEP	0.12	20	34.27	88.86	-0.18
	TPhP	0.11	9	30.79	94.42	-0.01
	DBUP/DiBP	0.15	9	10.53	34.53	0.98
	TBOEP	0.2	3	94.82	97.82	-0.35
	TnBP/TiBP	0.25	1	26.58	26.58	.
	DoCP	0.28	3	14.76	15.77	0.98
	TCEP	0.11	0	.	.	.
	TPP	0.31	0	.	.	.
Benzophenones	BP1	0.04	135	6.29	96.19	0.98
	BP3	0.05	135	15	98.57	0.92
	BP8	0.14	74	12.48	83.09	0.71
	OH4BP	0.17	33	16.74	56.04	0.76
	BP6	0.06	14	8.12	40.1	0.89
	BP2	0.22	5	10.16	28.37	0.79
Bisphenols	BPS	0.05	96	13.94	87.73	1
	BPF	0.05	74	18.66	88.86	0.92
	BPA	0.06	40	8.77	82.02	0.84
	BADGE	0.18	1	1.93	1.93	.
	BPAP	0.21	1	46.09	46.09	.
	BPP	0.06	0	.	.	.
	BADGE2H2O	0.63	2	22.15	41.89	0.38
	BPB	0.35	1	32.3	32.3	.
	BPZ	0.08	0	.	.	.
	BADGEH2O	0.68	1	18.18	18.18	.
	BPAF	0.04	0	.	.	.
	TBBPA3355	0.08	0	.	.	.
	TECBPA2266	0.26	0	.	.	.
	TrCBPA335	0.34	0	.	.	.
Parabens	MePB	0.03	136	6.25	78.77	0.96
	PrPB	0.03	131	5.96	86.31	0.99
	EtPB	0.07	120	7.78	40.43	0.98
	BuPB	0.1	44	14.75	46.94	0.94
	BzPB	0.05	29	4.37	27.93	0.96
	HePB	0.17	0	.	.	.
Antimicrobials	TCS	0.07	127	6.75	89.21	0.96
	TCC	0.05	53	16.89	84.09	0.85
Phthalates & Alternatives	MEHHP	0.07	135	7	78.04	0.94
	MEOHP	0.05	136	5.79	79.72	0.93
	MECPP	0.35	131	9.11	81.66	0.86

© 2026 Buckley JP et al. *JAMA Network Open.*

Chemical Class	Analyte (acronym)	LOD (ng/mL)	Valid Pairs	RPD Median	RPD Max	ICC
	MCMHP	0.18	100	19.92	70.53	0.7
	MEHP	0.09	91	15.58	83.69	0.91
	MCiOP	0.05	134	8.45	75.33	0.95
	MHiNP	0.12	80	9.29	70.97	0.97
	MPCHP	0.05	130	6.88	53.85	0.95
	MPOHP	0.04	86	9.87	73.43	0.92
	MPHHP	0.06	22	26.24	74.41	0.38
	MCiNP	0.06	128	13.2	62.43	0.96
	MHiDP	0.16	8	19.85	54.65	0.65
	MiDP	0.06	3	14.03	31.62	-0.23
	MBzP	0.08	131	9.53	78.94	0.93
	MCPP	0.06	113	12.91	74.27	0.97
	MCHpP	0.23	102	12.26	76.26	0.99
	MOP	0.07	2	14.56	23.34	0.03
	MHPP	0.05	85	14.58	92.12	0.97
	MHxP	0.04	79	17.65	56.04	0.79
	MPrP	0.11	9	10.45	52	0.72
	MPeP	0.04	1	13.56	13.56	.
	METP	0.08	1	9.03	9.03	.
	MtBTP	0.08	0	.	.	.
	MBzTP	0.09	0	.	.	.
	MEP	0.08	136	5.07	46.51	0.96
	MnBP/MiBP	0.15	136	6.12	66.45	0.91
	MMP	0.32	61	19.23	73.66	0.41
	MiPP	0.1	28	13.78	48.47	0.61
	MHiNCH	0.04	65	12.37	52.96	0.98
	MONCH	0.05	65	17.78	77.78	0.92
	MCOCH	0.06	41	21.76	65.61	0.84
	PA	5	10	21.1	99.09	-0.01
Polycyclic Aromatic Hydrocarbons	NAP2	54	135	10.97	88.8	0.94
	NAP1	110	101	28.89	83.16	0.8
	PHEN1/PHEN9	190	84	12.87	70.87	0.75
	PYR1	58	89	12.25	67.63	0.85
	PHEN2/PHEN3	110	65	9.87	39.55	0.87
	FLUO2/3/9	350	60	13.79	39.69	0.95
	PHEN4	150	17	10.96	50.14	0.56

Abbreviations: ECHO, Environmental influences on Child Health Outcomes; ICC, intraclass correlation coefficient; LOD, limit of detection; RPD, relative percent difference.

© 2026 Buckley JP et al. *JAMA Network Open.*

eReferences.

1. Pellizzari ED, Woodruff TJ, Boyles RR, et al. Identifying and prioritizing chemicals with uncertain burden of exposure: opportunities for biomonitoring and health-related research. *Environ Health Perspect*. Dec 2019;127(12):126001. doi:10.1289/EHP5133

2. Zhu H, Chinthakindi S, Kannan K. A method for the analysis of 121 multi-class environmental chemicals in urine by high-performance liquid chromatography-tandem mass spectrometry. *J Chromatogr A*. Jun 7 2021;1646:462146. doi:10.1016/j.chroma.2021.462146

3. Kannan K, Stathis A, Mazzella MJ, et al. Quality assurance and harmonization for targeted biomonitoring measurements of environmental organic chemicals across the Children's Health Exposure Analysis Resource laboratory network. *Int J Hyg Environ Health*. May 2021;234:113741. doi:10.1016/j.ijheh.2021.113741

4. Hornung RW, Reed LD. Estimation of average concentration in the presence of nondetectable values. *Appl Occup Environ Hyg*. 1990;5(1):46-51. doi:10.1080/1047322X.1990.10389587

© 2026 Buckley JP et al. *JAMA Network Open.*
