# Supplementary material for: Gestational Exposure to 10 Classes of Priority Chemicals and Birth Outcomes in the ECHO Cohort
Source: JAMA Netw Open. 2026 Jun 17;9(6):e2618883. doi: 10.1001/jamanetworkopen.2026.18883 (PMC13276631; doi:10.1001/jamanetworkopen.2026.18883)
Supplement: Supplement 4. — Data Sharing Statement [file jamanetwopen-e2618883-s004.pdf]

Data Sharing Statement

Buckley. Gestational Exposure to 10 Classes of Priority Chemicals and Birth Outcomes in the ECHO Cohort. *JAMA Netw Open*. Published June 17, 2026.
doi:10.1001/jamanetworkopen.2026.18883

Data
Data available: Yes
Data types: Deidentified participant data
How to access data: Select de-identified data from the ECHO Program are available through NICHD's Data and Specimen Hub (DASH). https://echochildren.org/dash/
When available: beginning date: 02-01-2025

Supporting Documents
Document types: None

Additional Information
Who can access the data: Data used for this analysis are publicly available by request via the ECHO study DASH webpage.
Types of analyses: Data are publicly available and can be requested by anyone.
Mechanisms of data availability: The data are available by request for download from the ECHO study DASH repository: https://echochildren.org/dash/.
